# Heterosis and combining ability in cytoplasmic male sterile and doubled haploid based *Brassica oleracea* progenies and prediction of heterosis using microsatellites

**DOI:** 10.1371/journal.pone.0210772

**Published:** 2019-08-19

**Authors:** Saurabh Singh, S. S. Dey, Reeta Bhatia, Raj Kumar, Kanika Sharma, T. K. Behera

**Affiliations:** 1 Division of Vegetable Science, ICAR-Indian Agricultural Research Institute, New Delhi, India; 2 Division of Floriculture and Landscaping, ICAR-Indian Agricultural Research Institute, New Delhi, India; 3 ICAR-Indian Agricultural Research Institute, Regional Station, Katrain, Kullu, Himachal Pradesh, India; New South Wales Department of Primary Industries, AUSTRALIA

## Abstract

In *Brassica oleracea*, heterosis is the most efficient tool providing impetus to hybrid vegetable industry. In this context, we presented the first report on identifying superior heterotic crosses for yield and commercial traits in cauliflower involving cytoplasmic male sterile (CMS) and doubled haploid (DH) lines as parents. We studied the suitability of genomic-SSRs and EST-SSRs based genetic distance (GD) and agronomic trait based phenotypic distance (PD) for predicting heterosis in F_1_ hybrids using CMS and DH based parents. 120 F_1_ hybrids derived from 20*Ogura* based CMS lines and 6 DH based testers were evaluated for 16 agronomic traits along with the 26 parental lines and 4 commercial standard checks. The genomic-SSRs and EST-SSRs based genetic structure analysis grouped the 26 parental lines into 4 distinct clusters. The CMS lines Ogu118-6A, Ogu33A, Ogu34-1A were good general combiner for developing early maturity hybrids. The SCA effects were significantly associated with heterosis suggesting non-additive gene effects for the heterotic response of hybrids. Less than unity value of σ^2^A/D coupled with σ^2^_gca_/σ^2^_sca_ indicated the predominance of non-additive gene action in the expression of studied traits. The correlation analysis of genetic distance with heterosis for commercial traits suggested that microsatellites based genetic distance estimates can be helpful in heterosis prediction to some extent.

## Introduction

In the plant kingdom, the family *Brassicaceae* holds a great agronomic, scientific and economic significance and comprises of more than 372 genera and 4060 species [1]. *Brassica oleracea* (CC, 2n = 18) constitutes a diverse group of economically and nutritionally important morphotypes known as cole vegetables (kale, kohlrabi, cabbage, cauliflower, broccoli, brussels sprout) [2]. The *Brassica* vegetables are also termed as ‘super-food’ as they are vital source of secondary metabolites, antioxidants, vitamins and minerals [3, 4, 5, 6]. Among the cultivated *B*. *oleracea* morphotypes, cauliflower (*B*. *oleracea* var. *botrytis* L.) is an important vegetable crop grown worldwide. Great efforts have been made to improve the productivity and quality of this crop because of its huge economic value and quality attributes [7]. The replacement of open-pollinated varieties with F_1_ hybrids become more pronounced in cole vegetables because of their high uniformity, better quality, tolerance to various biotic and abiotic stresses [5, 8]. The genetic mechanisms namely, sporophytic self-incompatibility (SI) and cytoplasmic male-sterility (CMS) have been used widely in hybrid breeding programme of *B*. *oleracea* [5, 8, 9, 10, 11, 12]. However, frequent breakdown of self-incompatibility has been reported in *Brassica* vegetables due to the high temperature sensitivity of S-alleles. Thus, SI lines are not always stable and result in ‘sibbed’ seed in hybrid population [11]. Moreover, maintenance of S-allele lines is time-consuming and expensive. In snowball cauliflower, SI system is very poor or not present at all [11, 13]. Under these circumstances, the CMS provides a better alternative for the heterosis breeding in cole crops [5, 8, 14].

Heterosis or hybrid vigor, is manifested as superior performance of F_1_ hybrids as compared to the parents [15, 16, 17]. Heterosis is highly complex phenomenon and different hypothesis and genetic basis have been suggested to explain the basis of heterosis. [15, 16, 18, 19, 20]. Further, the application of genomics tools has suggested the role of epigenetic regulations in explaining the heterosis phenomenon across the crops [16, 17, 19, 20]. Proper selection of inbreds and identification of superior heterotic combinations is crucial for exploiting heterosis in crop improvement. The traditional approaches of quantitative genetics like diallel, generation mean, line × tester analysis and estimating genetic components revealing various gene effects are effective in unraveling genetic basis of heterosis [5, 15, 21, 22]. The measures of both general combining ability (GCA) and specific combining ability (SCA) are necessary for selection of parental lines to develop heterotic combinations [23]. Estimation of GCA provides information on breeding value and additive genetic variance while, SCA is associated with non-additive effects (dominance effects, additive×dominant, and dominant×dominant interactions). Among different biometrical approaches, line × tester analysis is very efficient for estimating GCA effects of lines and testers, SCA effects of cross combinations and revealing information about the nature of gene actions [8, 21, 24]. The extent of heterosis has been reported to vary with the mode of reproduction, genetic distance of parents, traits under investigation, developmental stage of plant and prevailing environment [23, 25, 26, 27, 28, 29]. The pair-wise parental GD has been suggested as a good indicator of *per se* hybrid performance and recognition of heterotic groups [23, 25, 26, 27, 28, 29].

Different approaches are available to determine genetic distance depending upon morphological traits, horticultural data, biochemical characteristics and DNA markers based genotypic data [30, 31]. The SSR (simple sequence repeat) markers have been regarded as the markers of choice owing to their co-dominant inheritance, whole-genome coverage, abundance and high reproducibility [27, 32]. However, contradictory results have been reported with respect to the relationship between genetic distance and heterosis in different crops [15, 24, 25, 26, 27, 28, 29, 30, 33, 34]. According to Cress [35], the extent of parental genetic distance is essential but is not enough to assure the significant heterosis. It was also suggested that the better forecasting of heterosis is possible only when genetic distance is less than a definite threshold level [36]. The association of genetic distance and heterosis also depends upon the germplasm, population structure under investigation and methods of calculation.[29, 37]. The parents with lesser genetic distance can also display a high level of heterosis when closely related ecotypes of *Arabidopsis* are used to develop hybrids [16, 38]. Contrasting results are reported about relationship between phenotypic distance (PD), genetic distance (GD), heterosis and specific combining ability (SCA) in different crops [30, 34, 39, 40]. Teklewold and Becker [39] have reported significant positive association of PD with mid-parent heterosis (MPH), general combining ability (GCA) and hybrid performance for seed yield in Ethiopian mustard (*Brassica carinata*), while Hale et al. [26] found no correlation between PD and heterosis in broccoli.

The development of homozygous inbred lines is tedious and time consuming process in *B*. *oleracea* as these crops are highly heterozygous in nature [41]. Availability of completely homozygous inbred lines is pre-requisite in hybrid development. Development of inbreds through conventional approaches requires several generation selfing. Presence of high inbreeding depression makes the process of inbred development more complicated. On the other hand, development of doubled haploids (DHs) enables to produce large number of completely homozygous lines within a very short period of time. DH technology has been used widely in *B*. *oleracea* in different genetic and genomic research, such as QTL mapping, construction of high density genetic linkage maps and genome sequencing [41–51]. Our group has developed large number of DH lines on *Brassica* vegetables through isolated microspore culture [41, 52, 53]. The improved CMS lines of cauliflower have also been developed indigenously through protoplast fusion followed by recurrent backcrossing [9, 54]. Till date, only limited information is available regarding combining ability, gene action and heterosis using CMS systems and DH lines to improve yield and quality traits in *B*. *oleracea*. Moreover, no report is available in cauliflower regarding the association of GD with heterosis and combining ability for important traits. Several contrasting results have been reported in different crops in this context as heterosis is a complex biological phenomenon [16, 17, 19, 20]. Hence, the present investigation was conducted with the objectives (i) to identify heterotic combinations using CMS and DH lines and to analyze combining ability, nature of gene action and heritability for important traits. (ii) to find out correlation between microsatellites based genetic distance and morphological traits based phenotypic distance with heterosis and combining ability. The present investigation is the first report of heterosis and combining ability study based on CMS and DH lines in cauliflower.

## Materials and methods

### Plant materials

The field experiment was carried out at Baragram Experimental Farm of ICAR-Indian Agricultural Research Institute (IARI), Regional Station, Katrain, Kullu Valley, Himachal Pradesh, India. The experimental farm is located at 32.12N latitude and 77.13E longitudes with an altitude of 1,560 m above mean sea level. The basic plant material comprised of 20 genetically diverse *Ogura* CMS lines developed after more than nine generations of backcrossing (Table 1). These CMS lines were used as female parent in the breeding programme. Six DH based inbred lines of snowball cauliflower with abundant pollen production developed through isolated microspore culture (IMC) were used as testers (Table 1).

**Table 1:** Characteristics of parental lines (cytoplasmic male-sterile lines) and testers (doubled haploid lines) used in the study.

Code	Line	Curd Color	Curd compactness	Curd covering by inner leaves	Riceyness	Anthocyanin pigmentation	Origin of germplasm
L1	Ogu122-5A	White	Compact	PC	Absent	Absent	IARI, Katrain, India
L2	Ogu115-33A	White	Compact	PC	Absent	Absent	IARI, Katrain, India
L3	Ogu118-6A	White	Compact	PC	Absent	Absent	IARI, Katrain, India
L4	Ogu307-33A	Creamy White	Compact	NC	Absent	Absent	IARI, Katrain, India
L5	Ogu309-2A	Creamy White	Compact	PC	Absent	Absent	IARI, Katrain, India
L6	Ogu33A	White	Compact	FC	Absent	Absent	IARI, Katrain, India
L7	OguKt-2-6A	White	Compact	PC	Absent	Absent	IARI, Katrain, India
L8	Ogu1A	White	Compact	PC	Absent	Absent	IARI, Katrain, India
L9	Ogu13-85-6A	White	Compact	NC	Absent	Absent	IARI, Katrain, India
L10	Ogu1-6A	White	Compact	PC	Absent	Absent	IARI, Katrain, India
L11	Ogu2A	White	Compact	PC	Absent	Absent	IARI, Katrain, India
L12	OguKt-9-2A	White	Compact	PC	Absent	Absent	IARI, Katrain, India
L13	Ogu22-1A	Creamy White	Compact	PC	Absent	Absent	IARI, Katrain, India
L14	Ogu122-1A	White	Compact	PC	Absent	Absent	IARI, Katrain, India
L15	Ogu126-1A	White	Compact	PC	Absent	Absent	IARI, Katrain, India
L16	Ogu12A	White	Compact	PC	Absent	Absent	IARI, Katrain, India
L17	Ogu119-1A	Creamy White	Compact	PC	Absent	Absent	IARI, Katrain, India
L18	Ogu34-1A	White	Compact	PC	Absent	Absent	IARI, Katrain, India
L19	Ogu125-8A	White	Compact	FC	Absent	Absent	IARI, Katrain, India
L20	Ogu33-1A	Creamy White	Compact	PC	Absent	Absent	IARI, Katrain, India
T1	DH-18-8-1	White	Compact	PC	Absent	Absent	IARI, Katrain, India
T2	DH-18-8-3	White	Compact	PC	Absent	Absent	IARI, Katrain, India
T3	DH-53-1	White	Compact	FC	Absent	Absent	IARI, Katrain, India
T4	DH-53-6	White	Compact	FC	Absent	Absent	IARI, Katrain, India
T5	DH-53-9	White	Compact	PC	Absent	Absent	IARI, Katrain, India
T6	DH-53-10	White	Compact	PC	Absent	Absent	IARI, Katrain, India

L: lines, T: testers, PC: partly covered, FC: fully covered, NC: not covered

### Experimental design

All the recommended package of practices suggested for raising cauliflower crop at IARI- Regional station, Katrain, were followed to grow a healthy crop [55]. The size of the plot was kept 3.0 x 3.0 m^2^ with an inter and intra-row spacing of 45 cm. The 20 CMS lines of cauliflower were crossed with 6 DH male fertile testers to generate 120 testcross progenies by following the line × tester mating design [21]. To avoid any natural pollination, CMS lines were grown under net house. The fully opened flowers of CMS lines were pollinated with the freshly dehisced pollen from DH testers. The 120 testcross progenies along with their 26 parental lines were evaluated for various morphological, horticultural and yield -related traits along with 4 CMS based commercial hybrids (HVCF-18, HVCF-29, HVCF-16 from Acsen HyVeg and Pahuja from Pahuja Seeds) as standard checks. The 10×15 alpha lattice experimental design with three replications was used for conducting this study.

For recording the data on various agronomic traits, five well-established plants were randomly selected in each plot/block/replication.

### Phenotypic measurements

The 20 *Ogura* based CMS lines, 6 DH testers along with their 120 F_1_ hybrids were evaluated for sixteen phenotypic traits viz. (i) days to 50% curd initiation (CI); (ii) days to 50% curd maturity (CM); (iii) plant height(PH) (cm) (iv) gross plant weight(GPW) (g) (v) marketable curd weight (MCW); (g) (vi) net curd weight (NCW); (g) (vii) leaf length (LL) (cm); (viii) leaf width(LW) (cm); (ix) number of leaves (NoL); (x) curd length(CL) (cm); (xi) curd diameter(CD) (cm); (xii) core length (CoL) (cm); (xiii) curd size index (CSI) (cm^2^); (xiv) leaf size index (LSI) (cm^2^); (xv) harvest index (HI) (%); (xvi) Total marketable yield (TMY) (t/ha) [8–10, 56].

### DNA extraction and PCR amplification

The parental CMS lines and DH testers were grown in pro-trays under glasshouse conditions in a soilless mixture of cocopeat, perlite and vermiculite in the ratio of 3:1:1. The fully expanding leaves (100mg) from 25–30 days old seedlings were used for genomic DNA isolation using cetyltrimethyl ammonium bromide (CTAB) method with slight modifications [57]. The Genomic DNA samples were adjusted to 25–50 ng DNA/μl and the DNA samples were stored at -80°C till use. For the genetic diversity analysis in parental CMS and DH lines, 350 microsatellite primers (comprising of genomic-SSRs and EST-SSRs), distributed throughout C genome the *B*.*oleracea* [58, 59] were used. Out of 350 microsatellite primers, 145 primers were found to be polymorphic. However, only 87 SSRs and EST-SSRs which showed clear amplification and polymorphism were used for final molecular analysis. The PCR reaction mixture comprised of 1 μl of each forward and reverse primers, 2 μl of genomic DNA template (50 ng), 12.50 μl of 2× PCR Green master mix (GoTaq DNA polymerase; Promega, USA) and 8.50 μl nuclease free water. The Eppendorf Mastercycler Nexus GSX1 was used for PCR amplification. The PCR cycling programme was set up as follow: an initial denaturation of 94°C for 4 min followed by 35 cycles of denaturation at 94°C for 30s, annealing of primers at 50 to 60°C for 30s depending upon appropriate primer annealing temperatures and extension at 72°C for 1min, then final extension of 72°C for 7min. Amplified PCR products were separated by 3.0% agarose gel electrophoresis in 1X TBE buffer (pH 8.0) and the gel was run at 100 mA voltage for 120 min. Ethidium bromide (EtBr) of 0.5 mg/ml was used for gel staining and gel pictures were captured using digital gel documentation unit (BioSpectrum Imaging System, UK). The determination of fragment sizes was done using Promega 50 bp DNA step ladder.

### Genetic analysis

Among the 350 microsatellite markers, 87 polymorphic genomic-SSR and EST-SSRs loci depicting genetic diversity (S1 Table) were used for cluster analysis, Principal component analysis (PCA) and clustering through neighbor-joining (NJ) UPGMA method using DARwin software version 6.0.017 [60]. For testing the reliability of NJ dendrogram, a bootstrap value of 1000 replicates was used. For the allelic diversity analysis, estimation of observed number of alleles (N) per loci, observed heterozygosity (H_o_), expected heterozygosity (H_e_) and polymorphism information content (PIC) was computed through software CERVUS version 3.0 [61]. The estimation of PIC for each locus using CERVUS 3.0 was calculated according to the formula; PIC = 1-Ʃ P*i*^2^, where P*i* represents the *i*th allele frequency in a locus for the genotypes *P* under study [62].

The genetic structure analysis of the parental population of the testcross progenies was studied with Bayesian model-based clustering approach implemented in STRUCTURE version 2.3.4 software [63] to assign individuals to *k* clusters and sub-clusters. For the estimation of the proportion of ancestral contribution in each parental line, all simulations were performed by parameter setting as: “admixture model” with “correlated allele frequencies”. The algorithm was implemented with 10,000 lengths of the burn-in period followed by 100000 Markov Chain Monte Carlo (MCMC) repetitions and a plausible range of putative k values was kept from k = 1 to k = 10 run independently with 15 iterations for each k. The optimum value of k for determining most likely number of sub-populations was predicted according to the simulation method of DeltaK (*ΔK*) [64] with the help of web-based STRUCTURE HARVESTER version v0.6.94 [65].

### Statistical analysis

The agronomic data recorded for each parent, 120 F_1_ hybrids and 4 commercial checks in alpha lattice design were subjected to analysis of variance (ANOVA) using GLM procedure of SAS (statistical analysis system) software version 9.4 [66]. The line × tester statistical analysis of GCA, SCA, heterosis, heritability, variance and mean performance for was performed as per Kempthorne [21] through SAS version 9.4. The testing of significance of GCA and SCA effects was done at 5%, 1%, and 0.1% probability through F test. Heterosis estimates for different traits were computed as per Xie et al. [67] based on formulae: MPH% (Mid- parent heterosis) = [(F_1_-MP)/MP] x 100, BPH% (Better parent heterosis) = [(F_1_-BP)/BP] x 100, where MP is mid-parent and BP is better-parent performance and testing of significance was done at probability of p < 0.05, p < 0.01 and p < 0.01 through *F* test. The narrow-sense heritability (h^2^ns = V_A_/V_P_; V_P_ = V_G_ + V_E_) estimates were categorized into three classes viz., high (> 30%), medium (10–30%) and low (< 10%) [68]. The GA was calculated as = H^2^_b_× phenotypic standard deviation × K, where K value is 2.06, which is a standardized selection differential constant at 5% selection intensity [69]. The parental lines and testers were clustered into different groups based on sixteen agronomic traits using R software [70]. They were grouped through principal component analysis (PCA) to estimate the explained variance in the first two axes. Pooled data from five randomly selected plants of each genotype per plot per block per replication for all the sixteen morphological and commercial traits were taken for statistical analysis.

The Euclidean distance (ED), hereafter referred as phenotypic distance (PD) was calculated based on sixteen phenotypic traits using R software [70]. The simple matching dissimilarity coefficient (hereafter referred as genetic distance: GD) was computed based on g-SSR and EST-SSRs data analysis using DARwin software version 6.0.017. The association among genetic distances, heterosis and combining ability was computed by Pearson’s correlation coefficients (r) (pearson product moment correlation coefficient: PPMCC) by using R software packages version 3.5.1 in Rstudio 1.1.456 [70] and testing of significance at p < 0.05 and p < 0.01. The corrplot displaying correlation among distances, heterosis and combining ability was demonstrated via Rcorrplot package in Rstudio [71].

## Results

### Analysis of variance

The mean square estimates for different vegetative and commercial traits revealed significant differences among treatments for all the characters except curd diameter, core length and curd size index and curd diameter at 0.01% probability (S2 Table). Similarly, block effects were significant for all the traits except days to 50% curd initiation, curd diameter, core length and curd size index at the probability of 0.01% (S2 Table). The coefficient of determination (R^2^) indicated high variability percentage (>70%) for all the traits except curd length, curd diameter, core length and curd size index. The higher R^2^ value also suggests a higher significance of the model. The line × tester analysis of variance (ANOVA) for combining ability revealed highly significant differences among the treatments and parents for all the studied traits (Table 2). The mean squares of the lines were found significant for all the traits, while the mean squares for testers were for all except curd length (Table 2). The significant differences were also observed with respect to lines versus testers for all the traits except leaf width, curd length, curd diameter, core length, curd size index and harvest index, while the mean squares of parents versus crosses were significant for all the traits except for number of leaves (Table 2). The variance analysis for combining ability also revealed highly significant differences among the120 testcross progenies for all the 16 traits at 0.1% probability, while no significant differences were found among three replications for all the traits except leaf width, suggesting the presence of inherent variability among all the crosses (Table 2). The line × tester interaction effects were also significant for all 16 phenotypic traits.

**Table 2:** Line × Tester Analysis of variance (ANOVA) for combining ability for yield and horticultural traits in cauliflower.

**Source of variation**	**df**	**Days to 50% CI**	**Days to 50% CM**	**PH (cm)**	**GPW (g)**	**MCW (g)**	**NCW (g)**	**LL (cm)**	**LW (cm)**
Replicates	2	0.25	0.83	8.75	23153.58	11384.77	2565.82	1.65	6.04*
Treatments	145	6.08***	299.66***	176.18***	1095365.25***	266871.75***	106378.93***	175.89***	43.16***
Parents	25	13.28***	563.97***	113.34***	403231.69***	157665.16***	56107.28***	106.85***	29.26***
Parents (Line)	19	15.26***	598.47***	116.59***	324516.00***	129477.23***	47982.00***	104.48***	34.83***
Parents (Testers)	5	7.26***	35.52***	32.52*	93011.02***	92945.55***	59482.50***	72.38***	13.26***
Parents (L vs T)	1	5.75*	2550.53***	455.84***	3449932.75***	1016833.69***	193611.64***	324.21***	3.48
Parents vs Crosses	1	29.58***	1020.72***	5878.71***	31342492.00***	4855010.50***	1962991.75***	2546.75***	330.86***
Crosses	119	4.37***	238.08***	141.46***	986594.00***	251258.53***	101338.41***	170.48***	43.66***
Line Effect	19	5.53	1054.16***	222.35*	1737689.88*	335689.69	114123.88	270.94*	86.83**
Tester Effect	5	5.06	33.96	65.61	694204.25	153128.39	47422.77	180.44	49.83
Line * Tester Eff.	95	4.10***	85.61***	129.27***	851763.75***	239537.05***	101618.98***	149.86***	34.71***
Error	290	1.20	4.94	11.04	20306.63	10387.84	3296.66	4.67	1.96
Total	437	2.82	102.71	65.82	377032.50	95495.77	37496.81	61.47	15.65
** Source of variation**	**df**	**NoL**	**CL (cm)**	**CD (cm)**	**CoL (cm)**	**CSI (cm^2^)**	**LSI (cm^2^)**	**HI %**	**TMY (t/ha)**
Replicates	2	2.92	1.28	1.07	0.01	181.72	3407.57	6.81	18.22
Treatments	145	29.54***	2.35***	5.11***	1.23***	1325.33***	348863.53***	238.79***	426.99***
Parents	25	38.75***	1.07*	3.70***	0.95***	736.22***	169525.92***	305.09***	252.26***
Parents (Line)	19	40.96***	1.21*	4.14***	1.08***	820.98***	190813.19***	323.77***	207.16***
Parents (Testers)	5	15.33***	0.78	2.77***	0.61***	561.34**	88306.48***	292.54***	148.71***
Parents (L vs T)	1	113.78***	0.00	0.00	0.01	0.17	171165.05***	12.92	1626.93***
Parents vs Crosses	1	0.79	61.33***	168.99***	16.64***	43832.41***	3937259.75***	1887.12***	7768.02***
Crosses	119	27.84***	2.12***	4.03***	1.16***	1091.89***	356384.91***	211.01***	402.01***
Line Effect	19	33.27	2.20	3.84	1.39	1097.96	670563.13**	226.60	537.10
Tester Effect	5	23.30	0.10	3.10	0.58	264.71	413817.59	132.45	245.01
Line * Tester Eff.	95	27.00***	2.21***	4.11***	1.15***	1134.21***	290526.47***	212.03***	383.26***
Error	290	4.07	0.64	0.61	0.11	157.99	8677.53	27.14	16.62
Total	437	12.51	1.21	2.11	0.48	545.43	121529.77	97.27	152.79

^*, **, ***, ****^significant at 5%, 1%, 0.1%, 0.01% probability respectively through F test

### Genetic components of variance

The estimation of genetic components of variance revealed that nature of gene action, heritability, genetic advance and degree of dominance is presented in Table 3. The GCA variance (σ^2^_gca_) for both lines and testers was found lower as compared to the SCA variance (σ^2^_sca_) for all the studied traits except for Days to 50% curd maturity. The value of dominance variance (σ^2^D) was greater as compared to the additive component of variance (σ^2^A) for all the traits except for days to 50% curd maturity. The greater than unity value of degree of dominance for all the traits indicated dominant nature of these traits except for days to 50% curd maturity. The ratio of additive to dominance variance (σ^2^A/D) coupled with predictability ratio (σ^2^_gca_/σ^2^_sca_) was less than unity for all the traits suggesting preponderance of non-additive gene action. The estimation of heritability is associated with selection efficiency. We observed the lowest estimate of narrow-sense heritability (h^2^_ns_) for curd length (3.44%) and the highest h^2^_ns_ value was recorded for days to 50% curd maturity (49.21%). Generally, moderate level of h^2^_ns_ estimates was found for majority of the traits except for CL, CD, CSI, and HI. The higher estimates of genetic advance (GA) at 5% selection intensity were observed for GPW, MCW, NCW and LSI, while lower estimates of GA were recorded for all the remaining traits.

**Table 3:** Estimates of genetic components of variance, heritability, genetic advance and predictability ratio for sixteen vegetative and commercial traits.

Variance components	Days to 50% CI	Days to 50% CM	PH (cm)	GPW (g)	MCW (g)	NCW (g)	LL (cm)	LW (cm)	NoL	CL (cm)	CD (cm)	CoL (cm)	CSI (cm^2^)	LSI (cm^2^)	HI %	TMY (t/ha)
σ^2^_gca_line	0.24	58.29	11.74	95410.18	18072.32	6157.07	14.79	4.71	1.62	0.09	0.18	0.07	52.22	36771.42	11.08	28.92
σ^2^_gca_ tester	0.06	0.48	0.91	11231.63	2379.01	735.44	2.93	0.80	0.32	-0.01	0.04	0.01	1.78	6752.33	1.76	3.81
sl^2^ GCA (Average) HS	0.10	13.82	3.41	30657.45	6000.54	1986.58	5.67	1.70	0.62	0.01	0.07	0.02	13.42	13679.82	3.91	9.60
σ^2^_sca_	0.97	26.89	39.41	277152.38	76383.07	32774.11	48.40	10.92	7.64	0.52	1.17	0.34	325.41	93949.65	61.63	122.21
σ^2^A	0.21	27.65	6.82	61314.89	12001.09	3973.16	11.33	3.40	1.24	0.03	0.15	0.04	26.84	27359.63	7.81	19.20
σ^2^D	0.97	26.89	39.41	277152.38	76383.07	32774.11	48.40	10.92	7.64	0.52	1.17	0.34	325.41	93949.65	61.63	122.21
σ^2^A/D	0.22	1.03	0.17	0.22	0.16	0.12	0.23	0.31	0.16	0.05	0.13	0.13	0.08	0.29	0.13	0.16
Degree of Dominance	2.15	0.99	2.40	2.13	2.52	2.87	2.07	1.79	2.48	4.46	2.82	2.78	3.48	1.85	2.81	2.52
Heritability (Narrow Sense) %	13.31	49.21	13.66	17.76	13.07	10.50	18.49	22.73	12.13	3.44	9.67	10.48	6.63	22.03	9.96	13.07
Genetic Advance 5%	0.34	7.60	1.99	214.97	81.57	42.07	2.98	1.81	0.80	0.06	0.25	0.14	2.75	159.92	1.82	3.26
Predictability Ratio	0.18	0.51	0.15	0.18	0.14	0.11	0.19	0.24	0.14	0.05	0.11	0.11	0.08	0.23	0.11	0.14

σ^2^A = additive genetic variance, σ^2^D = dominance genetic variance, σ^2^_gca_ = estimate of GCA variance, σ^2^_sca_ = estimate of SCA variance

### Combining ability effects

The estimates of combining ability are effective for early generation selection of inbred lines and identifying heterotic crosses. The GCA estimates of parental lines and testers are summarized in Table 4 and Table 5. The GCA estimates revealed that the CMS lines Ogu118-6A, Ogu33A, Ogu34-1A and Ogu33-1A had significantly high GCA in desirable direction for traits related to earliness. The CMS lines Ogu307-33A, Ogu119-1A, Ogu125-8A and tester DH-53-10 also showed significantly high GCA for days to 50% curd maturity in desirable direction. For core length, the significantly high GCA in the desirable negative direction was observed in CMS lines Ogu122-5A, Ogu118-6A, Ogu1A, Ogu13-85-6A, Ogu1-6A, Ogu122-1A and tester DH-53-10. Among the 20 female parents, 6 and 9 numbers of CMS lines exhibited high GCA for plant height and gross plant weight, respectively. Similarly, 6 and 9 numbers of CMS lines were found to good combiner for the marketable curd weight and net curd weight\ respectively. Among the 20 female parents, 8 numbers of CMS line for plant height, 9 numbers of CMS lines for gross plant weight, 6 numbers of CMS lines for marketable curd weight and 8 numbers of CMS lines for net curd weight exhibited significantly high GCA in desirable direction. The results pertaining to SCA effects of 120 cross combinations are presented in S3 Table. Among the 120 hybrids, 9 and 28 crosses showed significantly negative SCA effects for earliness traits CI and CM, respectively (S3 Table).

**Table 4:** Estimates of general combining ability (GCA) effects of lines and testers.

Lines/testers	Days to 50% CI	Days to 50% CM	PH (cm)	GPW (g)	MCW (g)	NCW (g)	LL (cm)	LW (cm)
Ogu122-5A	0.73**	6.78***	-0.10	278.71***	213.54***	31.59*	0.14	1.06**
Ogu115-33A	0.62*	5.56***	-2.29**	330.54***	26.21	24.64	5.09***	1.59***
Ogu118-6A	-0.60*	-7.99***	0.68	274.38***	23.93	148.70***	1.19*	2.26***
Ogu307-33A	-0.49	-13.16***	-0.03	209.32***	11.21	-14.02	5.17***	2.61***
Ogu309-2A	0.17	6.39***	-5.51***	-267.13***	-5.07	77.64***	-4.70***	-1.80***
Ogu33A	-0.71**	-13.27***	0.74	402.88***	179.27***	50.42***	-1.23*	-0.24
OguKt-2-6A	-0.16	4.84***	7.55***	609.65***	223.04***	77.20***	6.84***	5.56***
Ogu1A	-0.27	4.73***	-2.50**	19.88	18.27	-27.74*	-0.79	0.39
Ogu13-85-6A	0.06	-0.77	-3.32***	64.04	-41.46	40.31**	-6.09***	-3.54***
Ogu1-6A	-0.44	5.95***	-0.32	-40.40	62.66**	66.81***	-5.78***	-2.02***
Ogu2A	0.56*	4.51***	-0.84	-266.40***	-159.57***	-68.63***	-2.00***	-1.74***
OguKt-9-2A	0.68**	8.34***	2.87***	-282.07***	-135.73***	-56.63***	-0.18	0.84*
Ogu22-1A	0.12	2.95***	3.36***	-109.96**	-39.34	-53.13***	-2.38***	-2.14***
Ogu122-1A	0.56*	6.34***	3.31***	-148.29***	-105.84***	-84.13***	4.09***	-0.22
Ogu126-1A	0.56*	4.01***	1.83*	76.99*	115.16***	67.81***	4.55***	0.65*
Ogu12A	0.56*	6.89***	-0.89	-459.68***	-131.51***	-60.47***	-3.57***	-1.59***
Ogu119-1A	-0.05	-8.05***	1.36	-339.13***	-206.07***	-115.13***	0.27	-0.81*
Ogu34-1A	-1.10***	-11.61***	1.70*	78.49*	25.99	-0.97	1.78***	1.05**
Ogu125-8A	-0.10	-2.99***	1.42	171.71***	170.43***	71.14***	2.30***	1.30***
Ogu33-1A	-0.71**	-9.44***	-9.03***	-603.51***	-245.12***	-175.41***	-4.70***	-3.22***
Testers								
DH-18-8-1	0.25	1.29***	-0.70	-145.43***	-67.08***	-39.01***	-2.78***	-1.39***
DH-18-8-3	0.16	0.28	-0.59	-27.38	-2.74	-25.86***	0.85***	0.25
DH-53-1	-0.40**	-0.36	1.38**	183.02***	78.32***	31.56***	2.12***	1.29***
DH-53-6	-0.34*	-0.72*	0.86*	-3.38	-35.79**	-3.36	-1.09***	-0.62***
DH-53-9	0.11	0.14	-1.35**	-38.20*	-1.61	11.89	0.95***	0.25
DH-53-10	0.21	-0.64*	0.40	31.37	28.91*	24.76***	-0.06	0.22
CD 95% GCA(Line)	0.51	1.03	1.54	66.17	47.32	26.66	1.00	0.65
CD 95% GCA(Tester)	0.28	0.57	0.84	36.24	25.92	14.60	0.55	0.36

^*, **, ***, ****^Significance at P ≤ 0.05, P ≤ 0.01, P ≤ 0.001, P ≤ 0.0001, respectively, CD: critical difference

**Table 5:** Estimates of general combining ability effects of lines and testers.

Lines/testers	NoL	CL (cm)	CD (cm)	CoL (cm)	CSI (cm^2^)	LSI (cm^2^)	HI %	TMY (t/ha)
Ogu122-5A	-0.48	-0.06	-0.17	-0.30***	-2.18	42.94	3.07*	8.54***
Ogu115-33A	0.79	0.15	0.18	0.26**	2.84	198.54***	-5.79***	1.05
Ogu118-6A	1.63***	0.05	-0.07	-0.23**	-1.02	132.96***	-5.21***	0.96
Ogu307-33A	0.29	-0.34	0.17	-0.07	-3.82	261.97***	-3.71**	0.45
Ogu309-2A	-1.54**	-0.41*	-0.72***	-0.05	-12.58***	-200.01***	6.05***	-0.20
Ogu33A	1.13*	0.33	0.01	0.00	3.38	-40.80	-1.78	7.17***
OguKt-2-6A	-0.15	0.21	0.38*	-0.15	5.62	445.65***	-4.05**	8.92***
Ogu1A	0.96*	0.31	0.48**	-0.48***	8.43**	-4.68	-0.23	0.73
Ogu13-85-6A	1.41**	-0.47*	-0.70***	-0.22**	-12.56***	-281.45***	-1.56	-1.66
Ogu1-6A	-2.43***	0.25	0.77***	-0.26**	9.57**	-231.80***	2.47*	2.51**
Ogu2A	1.91***	-0.12	0.20	-0.11	0.13	-143.18***	-2.02	-6.38***
OguKt-9-2A	-3.15***	0.35	-0.24	0.22**	2.37	28.93	-0.53	-5.43***
Ogu22-1A	-1.48**	-0.49*	-0.46*	0.70***	-3.55	-142.26***	1.93	-1.57
Ogu122-1A	-0.59	-0.09	-0.16	-0.22**	-2.54	79.39***	-0.97	-4.23***
Ogu126-1A	0.29	0.48*	0.25	0.15	7.58*	139.82***	3.39**	4.61***
Ogu12A	-0.65	-0.06	-0.22	0.23**	-3.34	-163.41***	5.69***	-5.26***
Ogu119-1A	0.96*	0.12	0.64***	0.26***	8.00**	-48.09*	-2.46*	-8.24***
Ogu34-1A	0.91	-0.34	-0.18	-0.06	-5.98*	103.02***	-1.81	1.04
Ogu125-8A	0.68	0.66***	0.66***	0.36***	14.24***	116.42***	3.52**	6.82***
Ogu33-1A	-0.48	-0.54**	-0.81***	-0.04	-14.60***	-293.94***	4.00**	-9.80***
Testers								
DH-18-8-1	-0.01	0.01	0.36***	0.17***	3.35*	-135.48***	1.04	-2.68***
DH-18-8-3	-0.98***	-0.07	-0.19	-0.07	-0.99	36.61**	0.58	-0.11
DH-53-1	0.14	0.03	0.16	-0.02	1.53	100.79***	-1.71*	3.13***
DH-53-6	0.02	0.03	-0.25*	0.00	-2.57	-54.16***	-1.87**	-1.43**
DH-53-9	0.97***	-0.02	-0.04	0.04	-0.77	39.02**	1.78**	-0.06
DH-53-10	-0.14	0.03	-0.05	-0.12**	-0.56	13.21	0.19	1.16*
CD 95% GCA(Line)	0.94	0.37	0.36	0.16	5.84	43.25	2.42	1.89
CD 95% GCA(Tester)	0.51	0.20	0.20	0.09	3.20	23.69	1.32	1.04

^*, **, ***, ****^Significance at P ≤ 0.05, P ≤ 0.01, P ≤ 0.001, P ≤ 0.0001, respectively, CD: critical difference

The highest SCA effect in the desirable negative direction for curd initiation was recorded in the hybrid Ogu307-33A × DH-18-8-3 followed by Ogu34-1A × DH-53-1 and Ogu309-2A × DH-53-9. For the days to 50% CM, the highest SCA effect in desirable negative direction was observed in the cross Ogu22-1A × DH-53-10 followed by Ogu125-8A × DH-53-6 and Ogu1A × DH-18-8-3. Among the 120 testcross progenies, 31 exhibited high significant positive SCA effects for PH. The highest positive significant SCA effect for PH was observed in the cross Ogu122-1A × DH-18-8-3 followed by Ogu13-85-6A × DH-53-1, Ogu13-85-6A × DH-18-8-3 and Ogu118-6A × DH-53-9. For the commercial traits viz. GPW, MCW, NCW, CL, CD, CoL and CSI, out of 120 crosses, 39, 32, 38, 10, 26, 33 and 24 crosses exhibited significantly high SCA effects in the desirable direction. For the vegetative traits, among 120 crosses, 44, 37, 33 and 48 crosses showed significantly high positive SCA effects for LL, LW, NoL and LSI, respectively. For the HI and TMY, among 120 hybrids, 18 and 32 hybrids displayed significantly high positive SCA effects, respectively. The cross combination Ogu22-1A × DH-53-6 exhibited highest positive significant SCA effect for GPW followed by Ogu307-33A × DH-18-8-3 and Ogu122-5A × DH-53-10. For the MCW, hybrid Ogu33A × DH53-1 showed the highest positive significant SCA effects followed by Ogu122-5A × DH-53-10 and Ogu1-6A × DH-53-1. The highly significant positive SCA estimate for NCW was observed in the cross Ogu33A × DH-53-1 followed by Ogu1A × DH-53-9 and Ogu22-1A × DH-53-6. For the total marketable yield, the highest significant SCA estimate in desirable positive direction was observed in the cross, Ogu33A × DH-53-1 (good combiner × good combiner) followed by Ogu122-5A × DH-53-10 (good combiner × good combiner) and Ogu1-6A × DH-53-1 (good combiner × good combiner) (S3 Table).

### Phenotypic characterization

**Fig 1:**
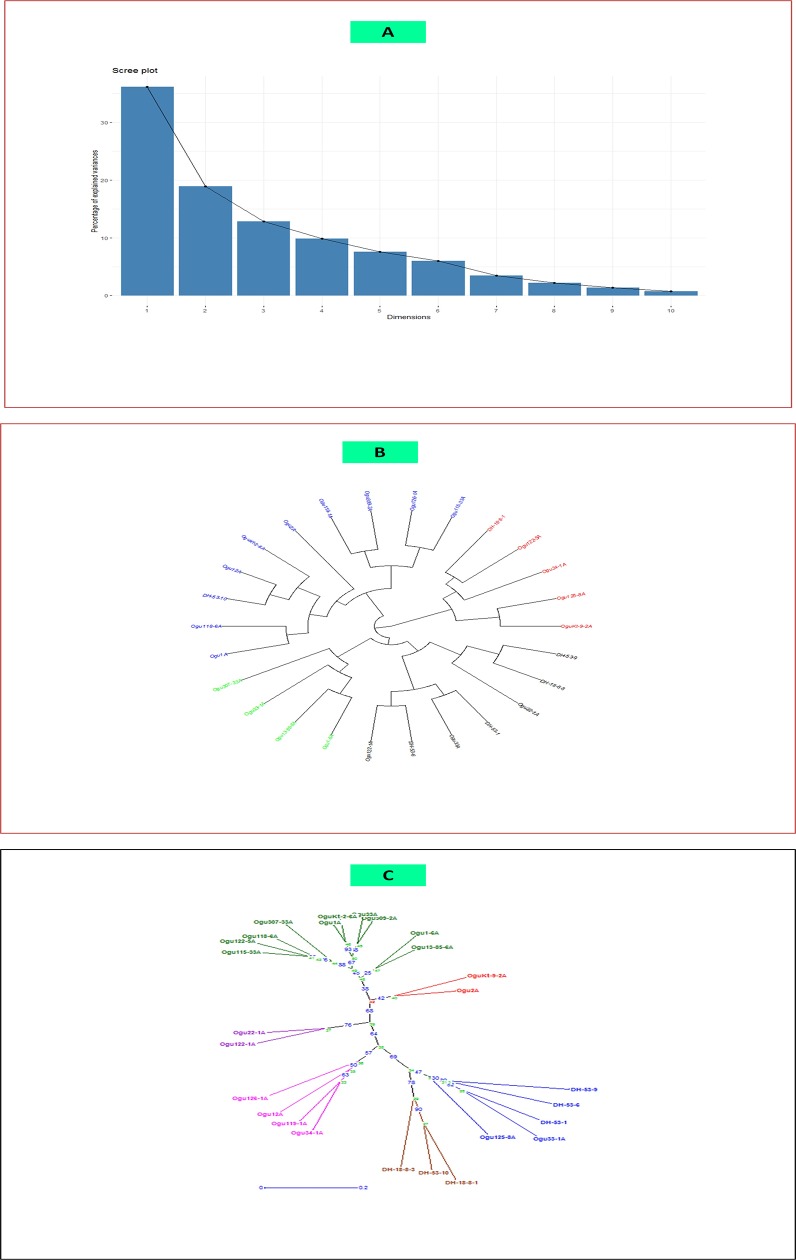
Morphological and molecular diversity in parental CMS and DH lines. (A) Percentage of explained variance among parental genotypes in different principal components (B) Dendrogram illustrating the genetic relationships among parental genotypes based on morphological traits (C) UPGMA cluster analysis illustrating the genetic relationships among parental genotypes based on g-SSR and EST-SSR analysis.

The mean performance of the parental CMS and DH lines along with standard checks is presented in S4 Table and S1A–S1H Fig. On the basis of curd initiation, the CMS lines Ogu307-33A and Ogu13-85-6A were earliest to initiate curd. Similarly, CMS lines Ogu307-33A and Ogu33-1A were earliest for curd maturity. The CMS line Ogu22-1A, OguKt-9-2A, Ogu12A and DH lines DH-53-1, DH-53-10 had the highest plant height. While CMS lines Ogu115-33A and Ogu309-2A were dwarf as compared to other parental lines. The highest number of leaves was observed in CMS lines Ogu34-1A and Ogu309-2A. The shortest core length was recorded in genotype Ogu13-85-6A and Ogu309-2A. The tester DH-53-1 was having highest MCW. The highest total marketable yield was recorded in CMS line Ogu33A and Ogu125-8A, whereas the tester DH-18-8-1 was having the highest TMY among all the parental genotypes and standard checks. The PCA revealed that the first two dimensions (PC1 and PC2) captured 36.2% and 18.9% of the total existing variation among the parental lines. The HCA based dendrogram depicting inter-relationships displayed high genetic divergence among 26 CMS and DH lines based upon Euclidean distance matrix (Fig 1B). On the basis of HCA, the 26 parental lines were classified into 3 major clusters with varying extent of divergence within internal sub-clusters. The DH lines DH-53-10 and DH-18-8-1 were distantly placed from the rest of DH testers in two different major clusters.

### Microsatellites based polymorphism, allelic diversity and genetic distances

Overall 511 alleles were amplified through 87 SSRs (Table 6) with the mean number of alleles per locus was 5.87. The allele numbers per locus ranged from 2 (1 primer BoSF1640) to 10 (1 primer: BRAS011). The observed heterozygosity (*H*_*o*_) ranged from 0.03 (for the loci BoSF2232, BoSF062, BRAS011, BoESSR080, BoSF2406 and BoSF2421) to 0.19 (for the loci BoSF2294a). The mean expected heterozygosity (*H*_*e*_) was 0.68, with a range of 0.27 (primer cnu107) to 0.83 (for primer Na12F03a and BoESSR041) and had a higher mean value than *H*_*o*_. The mean polymorphic information content (PIC) for 87 loci was 0.63. The PIC content ranged from 0.24 for the primer cnu107 to 0.80 for the primer Na12F03a (Table 6). Further, the PCA and Neighbour joining (NJ) cluster analysis based on molecular data for 87 loci, revealed distinct clusters and sub-clusters of parental CMS and DH lines based on their phylogeny (Fig 1C). The PCA revealed that the first two major coordinate axes 1 and 2 (PC1 and PC2) explained 61.41% of the total existing variation among CMS and DH lines. The dendrogram constructed revealed 3 main clusters of parental lines with internal sub-clusters showing varying degree of diversity. The DH testers remained in 2 different sub-clusters of the single main cluster. The CMS lines Ogu2A and OguKt-9-2A placed distantly from the rest of CMS lines. The CMS lines Ogu33-1A and Ogu125-8A were in close affinity with DH lines.

**Table 6:** Characteristics of 87 polymorphic SSR and EST-SSRs loci depicting diversity.

Locus	LG	H_o_	H_e_	N	PIC	Locus	LG	H_o_	H_e_	N	PIC
BoSF2304b	C09	0.00	0.48	5	0.43	BoESSR303	C04	0.00	0.33	4	0.31
BoSF1740	C08	0.00	0.63	5	0.57	BoESSR333	C04	0.00	0.81	9	0.77
BoSF378	C08	0.00	0.72	4	0.65	BoESSR338	C08	0.00	0.77	8	0.72
BoSF2680	C08	0.00	0.78	5	0.73	BoESSR403	C08	0.00	0.72	7	0.66
BoSF2054	C06	0.00	0.52	3	0.41	BoESSR409	C04	0.00	0.78	6	0.73
BoSF1215	C06	0.00	0.59	6	0.54	BoESSR576	C06	0.00	0.71	6	0.65
BoSF250	C06	0.00	0.67	3	0.58	BoESSR581	C06	0.00	0.67	7	0.62
BoSF2505	C06	0.00	0.60	4	0.54	BoESSR632	C01	0.00	0.71	6	0.67
BoSF2374	C05	0.00	0.62	4	0.54	BoESSR901	C09	0.00	0.77	7	0.73
BoSF1846	C05	0.00	0.69	4	0.62	BoESSR766	C03	0.00	0.69	7	0.63
BoSF2878	C05	0.00	0.57	3	0.48	BoESSR825		0.00	0.69	7	0.63
BoSF912	C01	0.00	0.72	6	0.67	BoESSR673	C03	0.00	0.66	5	0.58
BoSF063	C01	0.00	0.74	8	0.69	BoESSR758	C07	0.00	0.68	7	0.64
BoSF2294a	C02	0.19	0.73	6	0.67	BoESSR763	C04	0.00	0.80	8	0.76
BoSF2615	C02	0.00	0.61	4	0.55	BoESSR863	C06	0.00	0.69	4	0.62
BoSF1167	C02	0.00	0.79	6	0.75	BoESSR903	C06	0.00	0.50	5	0.46
BoSF2248	C03	0.00	0.60	5	0.55	Na12F03a	C07	0.07	0.83	8	0.80
BoSF2232	C03	0.03	0.49	3	0.38	O110B11	C05	0.00	0.65	4	0.57
BoSF184	C04	0.00	0.73	7	0.68	BoSF2406	C07	0.03	0.67	7	0.62
BoSF1640	C09	0.00	0.43	2	0.33	BoSF2313	C07	0.07	0.75	8	0.70
BoSF2612	C08	0.00	0.67	4	0.59	BoSF2033	C07	0.00	0.81	9	0.77
BoSF2860	C07	0.04	0.76	8	0.71	BoSF317	C05	0.00	0.70	6	0.66
BoSF2345	C01	0.00	0.76	5	0.71	BoSF2421	C09	0.03	0.61	6	0.57
BoSF1207	C01	0.07	0.77	8	0.73	BoSF1957	C04	0.00	0.82	8	0.77
BoSF042	C03	0.00	0.73	6	0.68	Na12B09	C03	0.00	0.70	6	0.66
BoSF062	C03	0.03	0.51	5	0.46	cnu107	C02	0.00	0.27	3	0.24
BoSF2985	C03	0.00	0.68	4	0.60	BoSF1131	C03	0.00	0.78	7	0.73
BoE862	C04	0.00	0.75	6	0.70	BoSF966	C03	0.00	0.80	7	0.75
BRAS011	C02	0.03	0.77	10	0.73	CB10258	C01	0.00	0.78	8	0.73
BrBAC214	C03	0.00	0.48	5	0.43	BoESSR920	C09	0.00	0.79	6	0.73
BoESSR080	C07	0.03	0.78	9	0.73	BoESSR041	C06	0.00	0.83	8	0.79
BoESSR086	C03	0.00	0.63	6	0.58	BoESSR934	C08	0.00	0.65	4	0.57
BoESSR087	C04	0.00	0.70	5	0.65	Ni4D12	C02	0.00	0.57	3	0.48
BoESSR089	C01	0.00	0.80	7	0.76	cnu149	C05	0.00	0.81	8	0.76
BoESSR105	C04	0.00	0.76	6	0.71	BoESSR482	C02	0.00	0.79	7	0.75
BoESSR108	C04	0.00	0.67	5	0.60	O112G04a	C08	0.00	0.44	4	0.40
BoESSR122	C02	0.00	0.82	8	0.78	BoESSR492	C03	0.00	0.76	8	0.72
BoESSR151	C02	0.00	0.70	6	0.65	BoESSR510	C03	0.00	0.59	4	0.51
BoESSR206	C05	0.00	0.53	4	0.47	BoESSR523	C07	0.00	0.76	7	0.71
BoESSR207	C05	0.00	0.78	5	0.72	BoESSR560	C03	0.00	0.73	6	0.67
BoESSR208	C04	0.00	0.71	8	0.66	BoESSR736	C05	0.00	0.66	5	0.61
BoESSR212	C07	0.00	0.56	4	0.51	BoESSR030	C03	0.00	0.76	8	0.71
BoESSR216	C01	0.00	0.76	5	0.70	BoESSR073	C03	0.00	0.50	6	0.46
BoESSR248	C04	0.00	0.62	5	0.57						

LG: linkage group, H_o_: observed heterozygosity, H_e_: expected heterozygosity, PIC: polymorphic information content

The Euclidean distance (PD) between lines and testers were computed from 16 phenotypic traits (S5 Table) and GD was calculated from molecular data based on 87 microsatellite markers (genomic-SSR and EST-SSRs) used for assessment of genetic diversity between parents (S5 Table). The PD was ranged from 2.07 for the cross L16 × T6 (Ogu12A × DH-53-10) to 8.27 for the cross combination L5 × T1 (Ogu309-2A × DH-18-8-1) with a mean of 5.52. The GD was ranged from 0.44 for the cross L20 × T1 (Ogu33-1A × DH-18-8-1) to 0.98 for the cross combinations L4 × T5 (Ogu307-33A × DH-53-9) with the average GD of 0.83.

### Genetic structure analysis

**Fig 2:**
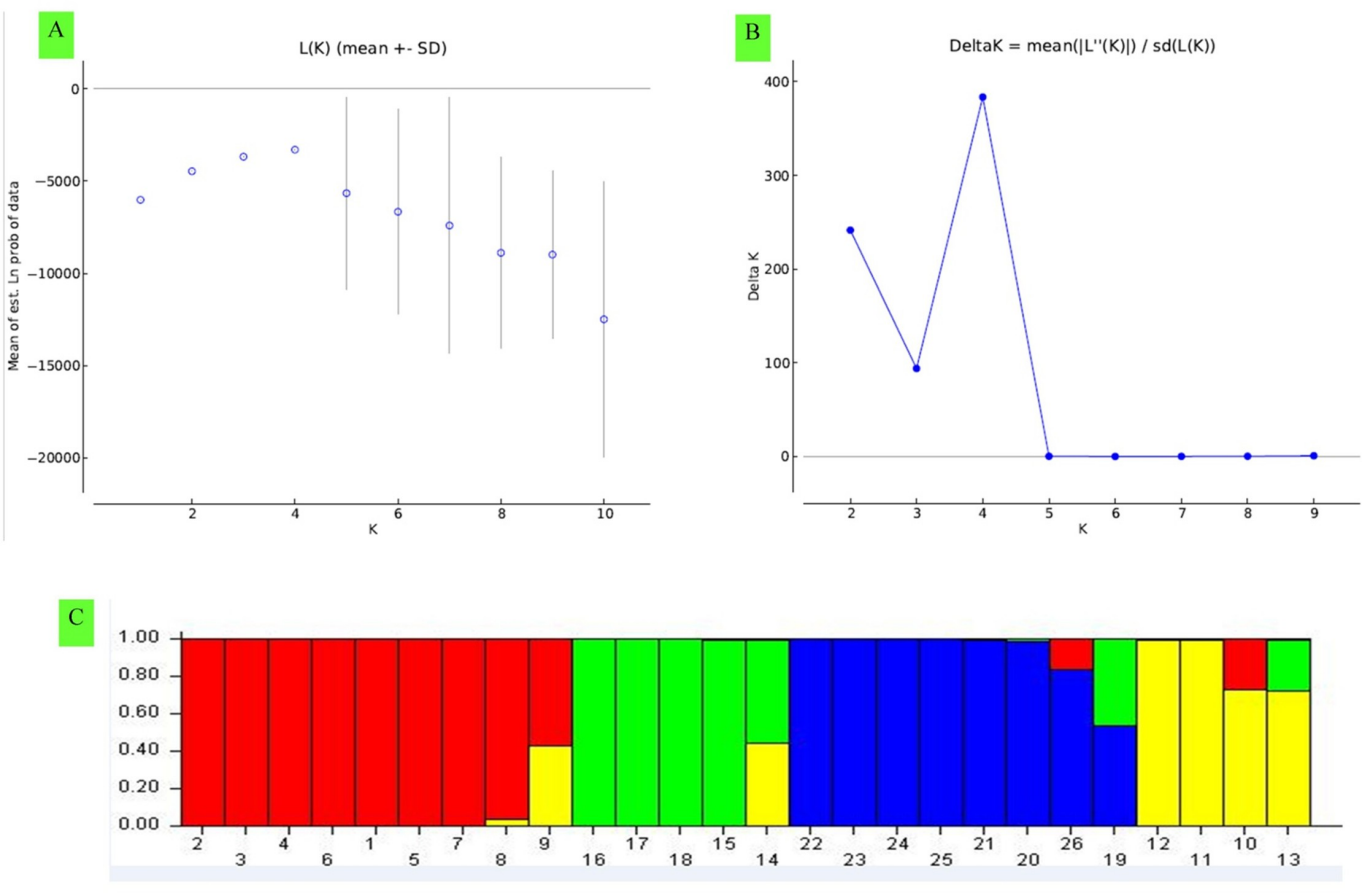
Genetic structure analysis of parental CMS and DH lines by STRUCTURE v2.3.4 and STRUCTURE HARVESTER based upon 87 g-SSR, EST-SSR loci. (a) Mean L(k) ± SD over 15 runs for each *k* value from 1 to 10. (b) *ΔK calculated as ΔK = m*|*L*′′ (*K*)|/*s* [*L*(*K*)], reached peak at *k* = 4. (c) Q-plot clustering. Inferred ancestries of CMS and DH lines based on 4 genetic groups. Each cluster is represented by different color and each column represent respective genotype allotted to respective cluster. Different color of each column depicts the percent of membership (vertical values on the left of cluster) of each genotype for four clusters.

The result of analysis by ‘Structure Harvester version v0.6.94’ revealed that second-order likelihood, *ΔK* reached to peak at *k* = 4 (Fig 2A to 2C), hence, optimal *k* value should be 4. This indicated that the 26 parental CMS and DH inbred lines could be grouped into 4 genetic sub-clusters (Fig 2C). All the DH testers placed in cluster III, along with 2 CMS lines Ogu125-8A and Ogu33-1A. Although there is minor admixture in DH-53-10 and Ogu125-8A from the genotypes of cluster I and cluster II, respectively. The other CMS lines placed themselves in separate clusters. The 20 parental CMS lines were grouped into 4 sub-clusters. The majority of the CMS lines were placed in cluster I. There is admixture from cluster IV to the cluster I (Ogu1A, Ogu13-85-6A) and cluster II (Ogu122-1A) genotypes. Similarly, there was minor admixture from cluster I and cluster II to cluster IV genotypes (Ogu1-6A and Ogu22-1A). Thus, there were four distinct four sub-clusters including minor gene flow within some genotypes of respective clusters from each other.

### Analysis of heterosis

The heterotic response of all the 120 testcross progenies varied in magnitude and highly significant heterosis was observed for all the 16 traits in both directions. The top ten cross combinations based on significant mid-parent heterosis in desirable direction along with their better-parent heterosis and SCA effects are presented in S6 and S7 Tables. The cross combinations, Ogu34-1A×DH-53-1 and Ogu33A×DH-53-6 showed significantly high MPH in the desirable direction for days to 50% curd initiation and days to 50% curd maturity (S6 Table). For CI, the testers DH-53-1 and DH-53-9 were involved in 4 crosses individually out of top 10 crosses. For CM the CMS line Ogu33A as a female parent was involved in 6 hybrids for earliness among top 10 hybrids. Ogu33A was also involved as female parent in one of the top 10 cross combinations related to CI. This line had significantly highest GCA for earliness among all the CMS lines used in the study. For the plant height, among the top 10 heterotic crosses, the cross combination Ogu118-6A×DH-53-9 exhibited highest significant positive heterosis over mid-parent followed by OguKt-2-6A×DH-53-9 and Ogu34-1A×DH-53-9. The highest significant positive heterosis for GPW was observed in the cross Ogu118-6A×DH-53-10 over mid-parent followed by Ogu126-1A×DH-53-1 and Ogu307-33A×DH-18-8-3. The top ten crosses having significant positive MPH for 8 commercial traits are presented in S7 Table.

The hybrid Ogu122-1A×DH-53-6 exhibited significantly highest MPH for CoL in desirable negative direction followed by Ogu1A×DH-53-6 and Ogu1A×DH-53-10. The CMS line Ogu1A was involved in 3 crosses as female parent among top 4 crosses with respect to core length, and it had significantly highest GCA in the desirable negative direction for core length. Thus, Ogu1A could be used as a parent for developing hybrids with short core.

For the commercial traits curd length and curd diameter, the cross combinations Ogu119-1A×DH-18-8-1 followed by Ogu126-1A×DH-53-1 and Ogu122-1A×DH-53-10 for CL, and Ogu1-6A×DH-53-1 followed by Ogu122-1A×DH-53-10 and Ogu22-1A×DH-53-6 for CD had highest MPH in the desirable positive direction. The cross Ogu122-1A×DH-53-10 showed the highest significant positive heterosis over mid-parent for CSI. For economic trait MCW, the hybrid Ogu126-1A×DH-18-8-3 followed by Ogu122-5A×DH-53-10 and Ogu307-33A×DH-18-8-3 exhibited significantly high positive MPH at P ≤ 0.001. Likewise, for the NCW, the significant highest heterosis over mid parent in the desirable positive direction was observed in the hybrid Ogu118-6A×DH-53-10 followed by Ogu1A×DH-53-9 and Ogu119-1A×DH-53-10. The cross combination Ogu122-5A×DH-53-9 (56.64%) showed highest significant MPH for percent HI followed by Ogu126-1A×DH-18-8-3 (49.16%) and Ogu12A×DH-18-8-3 (41.99%). For the total marketable yield, the highest significant heterosis over mid-parent in desirable positive direction was observed in the testcross Ogu126-1A×DH-18-8-3 followed by Ogu122-5A×DH-53-10 and Ogu307-33A×DH-18-8-3. Among the top ten crosses also wide range of MPH was recorded for TMY from 92.76% (Ogu33A×DH-53-1) to 172.31% (Ogu126-1A×DH-18-8-3).

### Association of genetic distances, heterosis and combining ability

**Fig 3:**
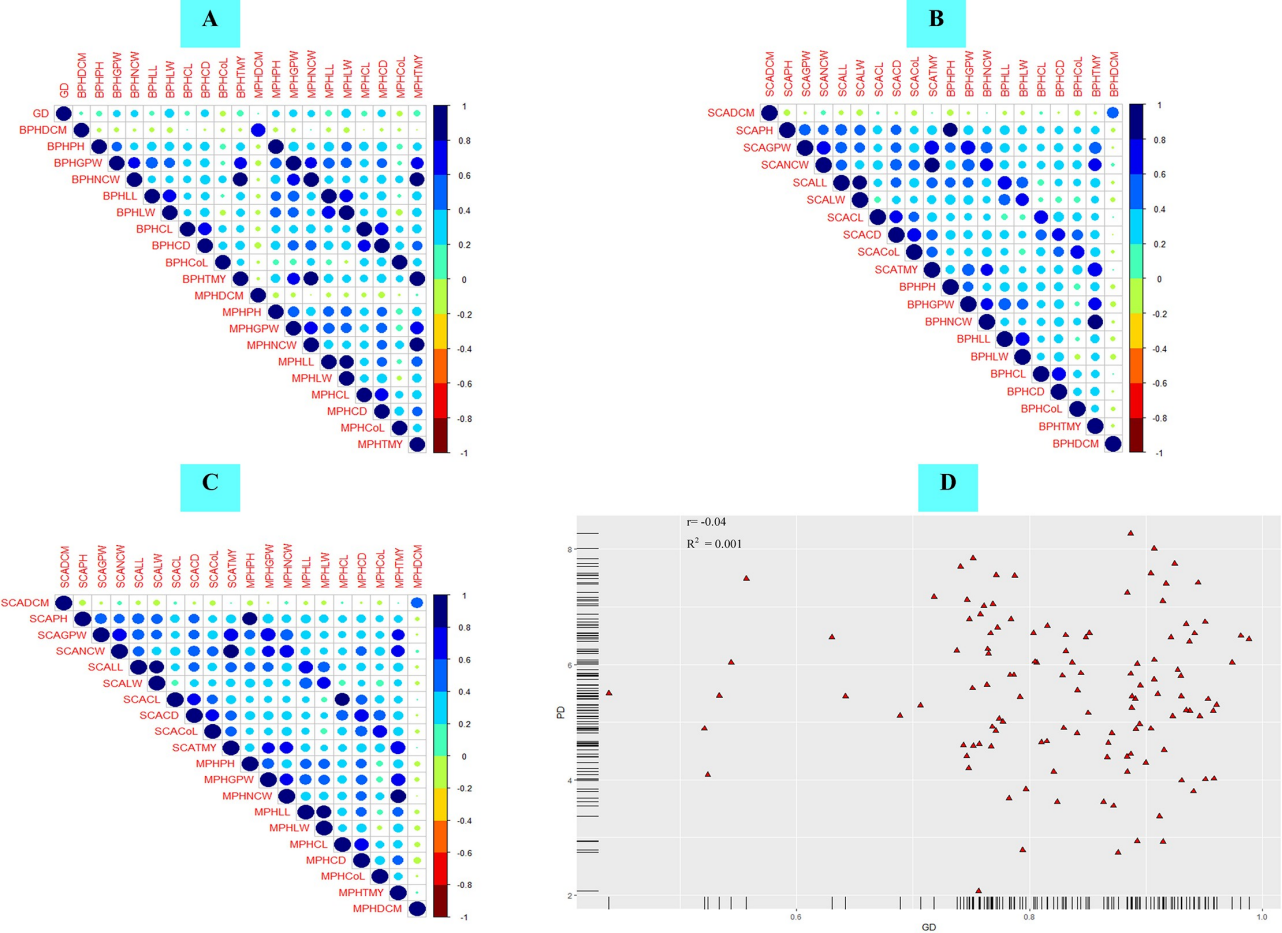
Pearson’s correlation matrix depicting association among genetic distances and heterosis. (A) Corrplot depicting pearson’s correlation coefficient of genetic distance based on molecular data with better-parent heterosis (BPH) and mid-parent heterosis (MPH) for 10 commercial traits, (B) Corrplot depicting pearson’s correlation coefficient of specific combining ability with BPH (C) Corrplot depicting pearson’s correlation coefficient of specific combining ability with MPH (D) Scatter plot depicting no correlation between genetic distance based on molecular data and phenotypic distance. GD is on X-axis and PD on Y-axis.

The Pearson correlation coefficient of genetic distances with heterosis, combining ability, and combining ability with heterosis for ten commercial traits is presented in Table 7. The GD and PD exhibited no significant correlation coefficient with SCA for any of the traits (Table 7 and S2B Fig, S2C Fig). SCA showed significantly positive correlation with MPH and BPH for all the traits at P ≤ 0.01 (Table 6, Fig 3B and 3C). No significant association of GD with MPH and BPH was observed with respect to days to 50% CM, leaf length, curd length and core length (Table 7, Fig 3A). For the commercial traits viz. plant height, gross plant weight, net curd weight, leaf width, curd diameter and total marketable yield, significant correlation was observed between GD and MPH in desirable direction for the respective traits (Table 7, Fig 3A). The highest magnitude of correlation coefficient of GD with MPH and BPH in desirable direction was observed for LW. However, PD exhibited no significant correlation with heterosis for majority of traits (Table 7, S2A Fig). The significant correlation of phenotypic distance with mid-parent heterosis was observed only for leaf length. PD exhibited a significant correlation in undesirable direction with core length. However, no significant association was observed between parental genetic distances based on phenotypic traits (PD) and molecular data (GD) (r = -0.04) (Fig 3D).

**Table 7:** Pearson correlation coefficients among parental genetic distance (GD, PD), combining ability and heterosis in cauliflower for ten morphological and commercial traits.

Traits	Days to 50% CM	PH	GPW	NCW	LL	LW	CL	CD	CoL	TMY
**GD**										
MPH	0.02	0.23*	0.27**	0.21*	0.17	0.34**	0.13	0.25**	-0.15	0.18*
BPH	0.07	0.15	0.21*	0.22*	0.12	0.36**	0.12	0.26**	-0.15	0.18*
SCA	-0.02	-0.01	0.02	-0.01	0.01	0.03	-0.01	0.01	0.03	0.01
**PD**										
MPH	0.12	0.14	0.07	-0.07	0.22*	0.14	0.01	-0.04	0.26**	-0.04
BPH	0.11	0.01	-0.07	-0.20*	0.16	0.07	-0.07	-0.14	0.24**	-0.25**
SCA	0.06	0.11	0.02	-0.05	0.09	0.12	0.07	0.02	0.03	-0.06
**SCA**										
MPH	0.52**	0.83**	0.79**	0.74**	0.73**	0.68**	0.81**	0.75**	0.74**	0.77**
BPH	0.53**	0.82**	0.78**	0.69**	0.69**	0.63**	0.74**	0.65**	0.69**	0.76**

^*, **^significance at P < 0.05 and P < 0.01, respectively, GD: genetic distance, PD: phenotypic distance, MPH: mid parent heterosis, BPH: better parent heterosis, SCA: specific combining ability

## Discussion

### Genetic components of variance and combining ability

The analysis of variance depicted highly significant differences among all the treatments for all the 16 agronomic traits, indicating considerable genetic differences among parents and their testcross progenies. The success of any crop breeding programme relies on genetic variation present in the germplasm. High genetic divergence among genotypes was reported by Garg and Lal [72], Verma and Kalia [73] for yield and related traits in early maturity Indian cauliflower, and for antioxidant traits in snowball cauliflower [5]. All the studied traits were found to be under the genetic control of both additive and non-additive gene effects, as revealed by the significant mean squares of lines, testers and line × tester interactions. The results are in agreement with Singh et al. [5] for antioxidant traits in cauliflower and Verma and Kalia [73] for days to 50% CM, leaf area, PH, MCW, NCW, curd compactness, GPW and HI in cauliflower using SI inbred lines. The analysis of genetic components of variance indicated the importance of SCA in developing heterotic crosses as revealed by the higher value of σ^2^_sca_ than σ^2^_gca_ of lines and testers for the majority of traits. As the response to natural and artificial selection relies on additive genetic variance, the narrow sense heritability (h^2^_ns_) holds a great promise in plant breeding as it provides the basis to precise selection of genotypes based on phenotypic variance because of additive genetic components [74]. In this study, the low to intermediate level of h^2^_ns_ was observed for majority of vegetative and commercial traits suggesting non-additive genetic control of these traits, which might be due to large epistatic effects. We also observed moderate estimates of h^2^_ns_ for antioxidant traits in cauliflower in our previous study [5], the results are also in agreement with Xie et al. [67] for mineral content in Chinese cabbage. Thus, the early generation selection for these vegetative and commercial traits would be difficult due to dominance effects in the expression of phenotypic variance, and hence selection must be practiced in later generations. The combining ability analysis has been successfully utilized in crop breeding for evaluating parental performance and understanding the dynamics of genes involved in trait expression. The parental GCA estimates in desirable direction also indicate potentiality of parents in generating promising breeding populations. In the present investigation, the significantly high GCA effects of parental lines in desirable direction for the respective vegetative and commercial traits are due to the predominance of additive genetic effects of genes and additive × additive interactions [5, 10]. It depicts a desirable gene flow from parents to progeny at high frequency and these parental lines exhibiting significantly high GCA for the respective traits in desirable direction can be utilized to accumulate favorable alleles via recombination and selection [5, 9, 10, 75, 76]. Further, our results revealed that none of the parents was good general combiner for all the studied vegetative and commercial traits. Similar findings were also reported inself-incompatible (SI) and CMS lines in cauliflower for yield and quality traits [5, 9, 73]. The findings indicate requirement of multiple breeding programmes in suitable mating designs for the development of productive cultivars with the accumulation of positive alleles of genes. On the other hand, the parental lines depicting GCA in opposite direction for the respective traits can be utilized to generate desirable mapping population to study the genetics of respective traits [76]. The SCA, which reflects the loci having non-additive and epistatic gene effects, can be utilized to determine specific heterotic crosses for the respective trait of interest. The inconsistent association of GCA and SCA of respective crosses for respective traits is the indication of complex interaction of genes for quantitative traits [29]. The majority of testcross progenies manifesting significantly high SCA in desirable direction had at least one of the parents reflecting poor GCA effects (poor × good general combiner or good × poor general combiner). It may be attributed to good combiner parent depicting favorable additive effects and poor combiner parent displaying epistatic effects [5, 77]. Few crosses with significantly high SCA in desirable direction for various traits had both parents with good GCA. (Such results suggested the role of cumulative effects of additive × additive interaction of positive alleles [5, 77]. Concurrently, some of the crosses had poor SCA effects for the respective traits, despite involving parents with significant GCA. It might be ascribed to the absence of any interaction among the positive alleles of genes. Thus, our results have clearly indicated that breeders must pay attention to both GCA and SCA in the selection of elite parents for the development of heterotic hybrids. Further, the recombination breeding and random mating in conjunction with selection among segregates (recurrent selection), synthetics and composites, may be exploited to harness utility of both additive and non-additive gene effects in cauliflower [73]. The high SCA effects are not always correlated with significantly high heterosis and concurrently, the heterotic crosses exhibiting high mid parent and better parent heterosis.

### Cluster analysis, allelic diversity, and genetic structure

The morphological and molecular diversity is vital in selecting desirable parents for hybrid breeding. The identification of heterotic groups and analyzing existing genetic variation in CMS lines is the preliminary requisite for efficient use of elite CMS lines in heterosis breeding. Study of genetic diversity at morphological and molecular level has been regarded as potential tool in identification of promising parental lines for developing heterotic hybrids in *B*.*oleracea* [8, 78–80]. Based upon PCA and HCA of 26 parental lines for 16 phenotypic traits, it was evident that all the parental lines had sufficient genetic variation. The PCA and NJ clustering based on molecular data represent better informative results for correct analysis and to be useful in crop improvement programme [8]. The female parents with significantly high GCA in the desirable direction for the traits related to earliness and high could be useful in developing early maturity and high yielding hybrids. Thus, the information pertaining to morphological and genetic diversity along with GCA could be useful in selecting desirable CMS lines as female parent for the development of cultivars with desirable traits.

We also observed high allele frequency of overall 511 alleles through 87 genomic-SSR and EST-SSRs loci in 26 parental CMS and DH lines with an average allelic frequency of 5.87 alleles per locus. Allelic diversity could be due to variation in germplasm used in the study, difference in methods of detection of markers and number of markers from transcribed regions of genome etc. [78, 79, 81, 82]. Varied allele frequency has been reported by various workers in *Brassica* crops with different set of germplasm and molecular markers [78, 79]. The PIC in genetic studies is utilized as a measure of informativeness of a marker locus for linkage analysis [78, 83] and it categorizes informative markers as highly informative (PIC ≥ 0.5), reasonably informative (0.5 < PIC >0.25) and slightly informative (PIC < 0.25) [78, 83]. In the present study the PIC content of 87 polymorphic loci ranged from 0.24–0.80, which classified all the 87 loci (g-SSR and EST-SSRs) as slightly informative (1 primer cnu107), reasonably informative (12 primers) and highly informative markers (74 primers). The mean PIC content of 0.63 in present investigation based on 87 genomic-SSR and EST-SSRs was higher than the mean PIC of 0.316 observed for 165 cauliflower inbred lines by Zhu et al. [84] and 0.60 as recorded for 57 genotypes of *Brassica oleracea* comprising 51 cultivars of cauliflower by Zhao et al. [85]. The DH lines developed through microspore culture are very diverse from most of the CMS lines. Therefore, these DH lines will be instrumental in developing heterotic combinations in snowball cauliflowers where genetic base is very narrow. Moreover, the diverse DH lines indicated the role of DH technology in creation of more diversity in different plant species. In all the clusters minor admixture was observed from each of clusters among themselves, which indicated the gene flow among the parental lines of different groups.

### Association of genetic distances and combining ability with heterosis

Numerous studies in different crops have revealed the utility of the genetic distances in prediction of heterotic crosses [23, 25–29], assuming positive correlation of genetic distances with heterosis [86]. But the correlation between GD and heterosis is not absolute and significantly high level of heterosis may result when parents with low, intermediate or high genetic distance are used. Genetic distances based on both phenotypic and genotypic data are utilized to study the genetic variation among different genotypes or parental inbred lines. The contrasting reports are available regarding correlations of genetic distances, heterosis and combining ability. In the present study, no correlation was observed between two distance measurements, based on morphological data (PD) and molecular data (GD). This is in contrary to the findings of Gupta et al. [30] who reported a significantly positive correlation of GD and PD (r = 0.2) at P < 0.001 in pearl millets. Absence of correlation between two distance measures might be due to the fact that morphological traits showing continuous variation are largely influenced by environment and polygenic inheritance besides linkage disequilibrium [30, 87–89]. Both the distance measures displayed no significant correlation with SCA of all the traits, suggesting genetic distances might not be effective in predicting SCA effects. Su et al. [29] also reported no significant association between genetic distances and SCA in chrysanthemum. However, Tian et al. [34], Lariepe et al. [28] reported a significant correlation between total GD and SCA for the length of terminal raceme in rapeseed and for grain yield and plant height in maize, respectively. Thus, the association of GD with SCA is not absolute. Further, our results suggested that SCA effects had a stronger significant positive correlation with MPH and BPH for all the studied traits. Similar findings were reported by Zhang et al. [90], Su et al. [29], Tian et al. [34] in barley, chrysanthemum and rapeseed, respectively they indicated non-additive gene effects for heterosis. The GD and PD differed in their ability to predict MPH and BPH for different traits. Neither GD nor PD displayed any significant correlation with MPH and BPH for days to 50% CM, and CL. GD also exhibited no significant correlation with heterosis for LL and CoL. Similarly, PD showed no significant association with heterosis for majority of traits except LL. However, GD was significantly correlated with MPH and BPH for important commercial traits viz. PH, GPW, NCW, LW, CD, and TMY in the desirable direction. These results are in line with the theory proposed by Falconer and Mackay [86]. In general, GD had a greater magnitude of correlation than PD with heterosis for all the traits under study. The variability in correlation coefficients between heterosis for respective traits and genetic distances may reflect allele numbers controlling the trait expression [26]. Similar findings were reported in Maize by Wegary et al. [89] where they have highlighted the importance of GD in contrast to PD for predicting hybrid performance. They have also reported a significant correlation of GD with heterosis for grain yield, plant height and ear height and morphological distance with heterosis for certain traits in quality protein maize. Jarosz [33] has reported a significant association of GD (based on RAP and AFLP markers) with heterosis for total and marketable yield in carrot. On the other hand, Tian et al. [34] and Su et al. [29] reported no significant correlation of PD and GD with MPH and BPH for any traits in rapeseed and chrysanthemum, respectively. Our results are also in contrary to the findings of Geleta et al. [91] and Kawamura et al. [27] in pepper and Chinese cabbage, respectively, suggesting no utility of GD in prediction of heterosis. While Krishnamurthy et al. [92] suggested selection of parents with intermediate divergence based on AFLP markers for getting a greater number of heterotic hybrids using CMS lines for yield in the hot pepper. Incole group of vegetables, we found only a single report describing interrelationships between genetic distances and heterosis in broccoli (*B*. *oleracea* var. *italic* L.) by Hale et al. [26] using DH based population. They observed a significantly negative correlation between total GD (based on SRAP, AFLP, SSR markers) and heterosis for all the traits, suggesting the reduction in heterosis with the increase in genetic distances. Thus, our study is the first comprehensive report regarding interrelationships between GD (based on SSR, EST-SSRs) and heterosis for commercial traits in snowball cauliflower, suggesting significant correlation in desirable direction for important yield related traits. Hence, based on this study, we recommend the application of genomic-SSR and EST-SSRs based genetic distances in prediction of heterosis for yield and commercial traits. The non-significant or poor correlation between GD and heterosis for certain traits might be due to the lack of linkage between different alleles responsible for expression of particular trait and molecular marker used for estimating GD, inadequate coverage of entire genome and epistasis. Besides, lack of correlation may also be due to use of DNA markers from unexpressed region of genome having no interaction with commercial traits and heterosis [26, 40, 62, 93]. The molecular marker-based GD would be more predictive of heterosis, when there are strong dominance effects among hybrids, high heritability, linkage of molecular markers and QTLs of traits of interest [26, 40, 62, 93]. Hence, from our findings, it is quite evident that significance of genetic distances in prediction of heterosis inevitably depends upon the methods used to calculate genetic distances, type of molecular markers, genome coverage, region of genome, crop, breeding system, traits under consideration, type of germplasm and environmental conditions. High correlation among the GD and heterosis among important traits may also because of completely homozygous DH lines as male parent. Presence of minor heterozygosity among the conventionally developed inbreds also results in poor correlation among GD and heterosis in most of the earlier study.

## Conclusions

The current study is first of its kind in determining the heterotic groups based on combining ability for morphological, yield and commercial traits using *Ogura* cytoplasm-based CMS lines and DH based testers. We also presented the first comprehensive report on predicting the association of genomic SSRs and EST-SSRs based GD and morphological traits-based PD with heterosis involving CMS and DH parental lines in snowball cauliflower. Analysis of variance of parents and their testcross progenies revealed the presence of significant genetic variability. Present investigation also emphasizes the relevance of both GCA and SCA in the selection of elite parents for the improvement of yield and commercial traits and predicting appropriate breeding strategies for the crop genetic improvement, developing high yielding hybrids, synthetics and composites in cauliflower. High and significant correlation among SCA with heterosis suggested the role of non-additive gene effects in heterosis. It was also evident that development of DH lines could broaden the genetic base of any crop through creating more diversity in the existing population. The findings of our study further suggested that genetic distances based on genomic and EST-SSRs can be used as a predictor of heterosis for commercial traits in CMS and DH based F_1_cauliflower. Contrasting results obtained in different earlier studies regarding the efficacy of genetic distances in the prediction of heterosis, invites further investigation with different sets of large number of molecular markers covering entire genome, and a different set of parental germplasm, in multiple standard environments.

## Supporting information

S1 Fig Boxplots for performance of parental genotypes for 16 phenotypic raits. DCI: days to 50% curd initiation, DCM: days to 50% curd maturity, PH: plant height, LL: leaf length, LW: leaf width, GPW: gross plant weight, MCW: marketable curd weight, NCW: net curd weight, CSI: curd size index, NoL: number of leaves, HI: harvest index, TMY: total marketable yield, CL: curd length, CD: curd diameter, CoL: core length, LSI: leaf size index. The upper and lower lines outside the box stand for maximum and minimum adjacent value, respectively. The median value is represented by line inside the box. The lower and upper hinge of the box stands for 25% and 75% percentile, respectively.
(TIF)

S2 Fig Pearson correlation coefficient of phenotypic distance, genetic distance and combining ability. (A) Corrplot depicting correlation of phenotypic distance with heterosis, (B) Corrplot of association of genetic distance with combining ability, (C) Corrplot depicting association of phenotypic distance with combining ability.
(TIF)

S1 Table List of 87 polymorphic genomic-SSR and EST-SSRs used for molecular diversity analysis. Among the 350 microsatellites, 87 primers showed clear cut polymorphism and used for allelic diversity analysis.
(DOCX)

S2 Table Estimates of Mean Squares and R2 for vegetative and commercial traits in Alpha Lattice Design. * = significant at 5% probability, ** = significant at 1% probability, *** = significant at 0.1%, **** = significant at 0.01% probability through F test, Rep = Replication, Blk = Block, Trt = Treatment, Rep (Blk)_Adj_ = Rep (Blk) Adjustable Days to 50% CI = Days to 50% curd initiation, Days to 50%CM = Days to 50% curd maturity, PH = Plant height, GPW = Gross plant weight, MCW = marketable curd weight, NCW = net curd weight, LL = leaf length, LW = leaf width, NoL = No of leaves, CL = curd length, CD = curd diameter, CoL = core length, CSI = curd size index, LSI = leaf size index, HI = harvest index, TMY = total marketable yield, R^2^: coefficient of determination.
(DOCX)

S3 Table Estimates of SCA effects of 120 test cross progenies for yield and horticultural traits. * = significant at 5% probability, ** = significant at 1% probability, *** = significant at 0.1%, **** = significant at 0.01% probability through F test, CD = critical difference, Days to 50% CI = Days to 50% curd initiation, Days to 50%CM = Days to 50% curd maturity, PH = Plant height, GPW = Gross plant weight, MCW = marketable curd weight, NCW = net curd weight, LL = leaf length, LW = leaf width, NoL = No of leaves, CL = curd length, CD = curd diameter, CoL = core length, CSI = curd size index, LSI = leaf size index, HI = harvest index, TMY = total marketable yield.
(DOCX)

S4 Table Characterization of parental CMS and DH lines including commercial checks for 16 phenotypic traits. *commercial standard checks.
(DOCX)

S5 Table Estimates of phenotypic distance (PD), based on 16 phenotypic traits and genetic distance (GD), based on g-SSR, EST-SSRs molecular data, between parental lines and testers. L: CMS lines; T: DH testers.
(DOCX)

S6 Table MPH of top ten crosses along with their BPH, mean performance and SCA effects (value in parenthesis) for 8 vegetative traits. * = significant at 5% probability, ** = significant at 1% probability, *** = significant at 0.1%, **** = significant at 0.01% probability through F test, MPH: Mid parent heterosis, BPH: better parent heterosis, SCA: specific combining ability (value in parenthesis).
(DOCX)

S7 Table MPH of top ten crosses along with their better parent heterosis and SCA effects (value in parenthesis) for 8 commercial traits. * = significant at 5% probability, ** = significant at 1% probability, *** = significant at 0.1%, **** = significant at 0.01% probability through F test, MPH: Mid parent heterosis, BPH: better parent heterosis, SCA: specific combining ability (value in parenthesis).
(DOCX)
