# Supplementary figures and images for: Heterosis and combining ability in cytoplasmic male sterile and doubled haploid based Brassica oleracea progenies and prediction of heterosis using microsatellites
Source: PLoS One. 2019 Aug 19;14(8):e0210772. doi: 10.1371/journal.pone.0210772 (PMC6699688; doi:10.1371/journal.pone.0210772)

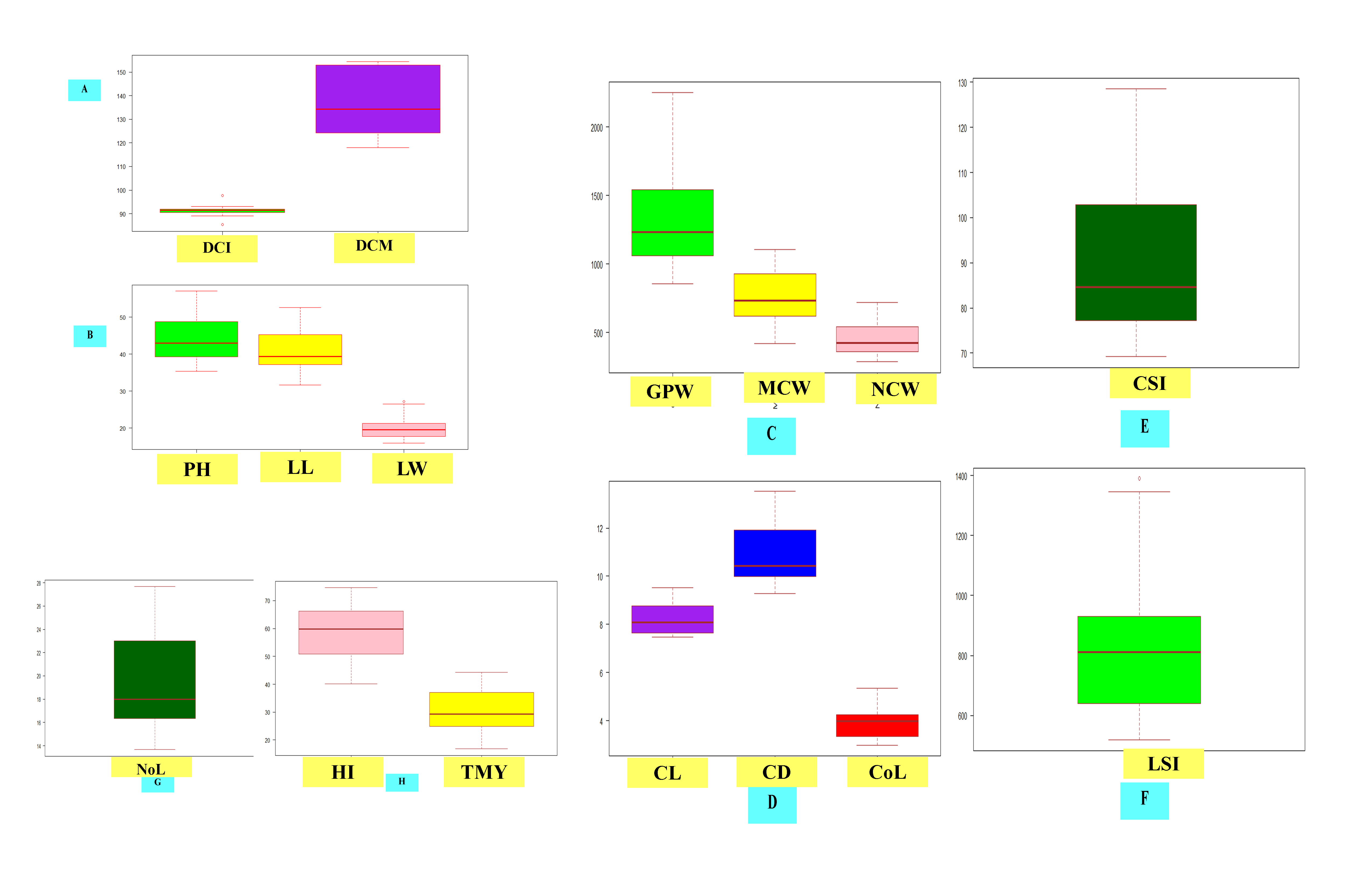

Supplement: S1 Fig — DCI: days to 50% curd initiation, DCM: days to 50% curd maturity, PH: plant height, LL: leaf length, LW: leaf width, GPW: gross plant weight, MCW: marketable curd weight, NCW: net curd weight, CSI: curd size index, NoL: number of leaves, HI: harvest index, TMY: total marketable yield, CL: curd length, CD: curd diameter, CoL: core length, LSI: leaf size index. The upper and lower lines outside the box stand for maximum and minimum adjacent value, respectively. The median value is represented by line inside the box. The lower and upper hinge of the box stands for 25% and 75% percentile, respectively. (TIF) [file pone.0210772.s001.tif]

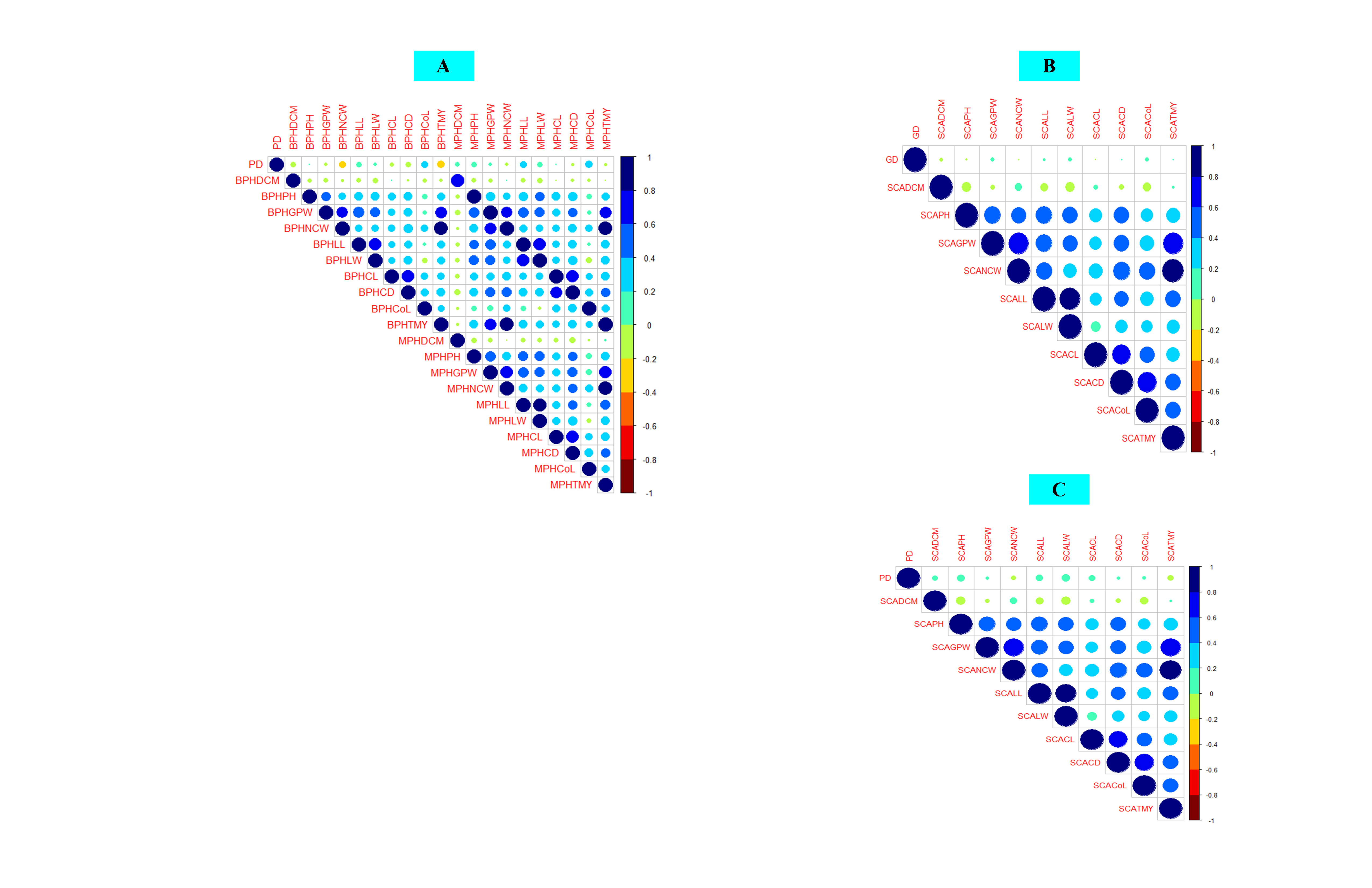

Supplement: S2 Fig — (A) Corrplot depicting correlation of phenotypic distance with heterosis, (B) Corrplot of association of genetic distance with combining ability, (C) Corrplot depicting association of phenotypic distance with combining ability. (TIF) [file pone.0210772.s002.tif]
