# Supplementary material for: Heterosis and combining ability in cytoplasmic male sterile and doubled haploid based Brassica oleracea progenies and prediction of heterosis using microsatellites
Source: PLoS One. 2019 Aug 19;14(8):e0210772. doi: 10.1371/journal.pone.0210772 (PMC6699688; doi:10.1371/journal.pone.0210772)
Supplement: S1 Table — Among the 350 microsatellites, 87 primers showed clear cut polymorphism and used for allelic diversity analysis. (DOCX) [file pone.0210772.s003.docx]

**S1 Table** List of 87 polymorphic genomic-SSR and EST-SSRs out of 350 microsatellite markers used for molecular diversity analysis

| **Sr. No** | **Marker Name** | **LG** | **Left Sequence** | **Right Sequence** |
| --- | --- | --- | --- | --- |
|  | BoSF2304b | C09 | AGAAACGCAAATGGTCTTCG | CTGTATGAGCATGGCTTCCA |
|  | BoSF1740 | C08 | AAGGCCAGTCCAACCAGAG | GATAACCGATCGGACATTCG |
|  | BoSF378 | C08 | AGTGTCCCTCAACTGCATCC | AAAGCTGAGTCACAGCCCAT |
|  | BoSF2680 | C08 | AAAGGTTAGGTGGTTGGATAAAGA | TGTCTTCTGATGCCTTGGTCT |
|  | BoSF2054 | C06 | GAAGGAACAAGAGGATGGCA | TCATGTTGTCGAGAATCCCA |
|  | BoSF1215 | C06 | AGTATCAAACCCGCCTGTTG | GGGTCGTATTAATCGCGTGT |
|  | BoSF250 | C06 | AAAAACACTTAGTTGTGGTGGGA | TTTTTAATGCAGCCCGAAAC |
|  | BoSF2505 | C06 | GGTTATTTCCACCATGCCAC | CTTGCCGAGACTCATCATCA |
|  | BoSF2374 | C05 | AAGCGGCACTCAACATAAGC | TAACCGTTTCTTGCTTGCCT |
|  | BoSF1846 | C05 | CCCTGCTTGAGATAAACCCA | CATGATGATGCCATACGCTC |
|  | BoSF2878 | C05 | CCTTGCGTCTGAAACATCAA | TTACCGGGAGTAAATGCAGC |
|  | BoSF912 | C01 | CTAGATCGCCTGCAAAGAGC | AATACGGGGAGGTAACTCGG |
|  | BoSF063 | C01 | GAATGTTTCCTCTGCTTGGC | TCAAAATCAGGAGAATCGGG |
|  | BoSF2294a | C02 | CACCATCGTTTCTGTCCCTT | TAACCACACCTTCCGTTTCC |
|  | BoSF2615 | C02 | CGTTGTCTCAAATCAATGGC | TCATCCATTATCATCGGGGT |
|  | BoSF1167 | C02 | TTCGTTCCTTCGTTCATTCC | AGGTAGTGGAGGAGGTCGGT |
|  | BoSF2248 | C03 | TCTGATAACCCCAGTTCCTTTT | ATTGTTGTCGATCGTGGTCA |
|  | BoSF2232 | C03 | CACTGCTCTACGCTCTCTCTTG | CCAAGTTGGGCCATACAGTT |
|  | BoSF184 | C04 | TTGCACGTACGTCTTTGAGG | CTGCAACGAGGATGAAAACA |
|  | BoSF1640 | C09 | AGCACAACTACCTGAACCTCT | TTTATCCTCGGTCTTCTCTCT |
|  | BoSF2612 | C08 | CGTAGCCGTCTCTTACGCAT | TTCAGTCCAGCGTTCAACAG |
|  | BoSF2860 | C07 | CATGCTTGCCTGAAAAGACA | CCTTGTACTGCTCCTCTGCC |
|  | BoSF2345 | C01 | GCTCCGATGATCACGATTCT | CTTCATCCCCTCACCACACT |
|  | BoSF1207 | C01 | CGACGCTAGACCAAGGTTTC | GGAAAACCTTCTGCCAATGA |
|  | BoSF042 | C03 | CGGCTTGACAGAATTGGACT | TCCTATTCCACACCAAAGCC |
|  | BoSF062 | C03 | CTAGTGTTCGCCGAAGTGGT | AAAAGGTGTCATGGAGTGCC |
|  | BoSF2985 | C03 | GGTTTCATAAAACATCTGTAGTTCGTC | TGCAAGACATCTTTATTTCTTCCTC |
|  | BoE862 | C04 | AGCAAAGGCGGGGGAATGATAC | ATGACAAAGACCACCCACACCAAT |
|  | BRAS011 | C02 | TGGGACGTAGTCAGTCAACAA | CCAAGTGCGAGAAGAGGAAG |
|  | BrBAC214 | C03 | CGTATAATTTTCATAGGCGACG | AGCATGCTTATGACTCTGGGA |
|  | BoESSR080 | C07 | GAACCGCTGAGGCAATTATG | CTTAGCGAGTCATGGGCTTC |
|  | BoESSR086 | C03 | ACCTCCAAACCATGACGAAG | GGTGGTGTTCTGTTGGCTTT |
|  | BoESSR087 | C04 | GTTTCCTCTTCCACCACCAA | AATCTATCAAGAGGGCCAAGG |
|  | BoESSR089 | C01 | ATGATCAGCGAAACCACTCC | TGATACATCCCGTTTGCTCA |
|  | BoESSR105 | C04 | GGAGAAGAGTCATGGCAAGG | GGAACGGCTCACTTCTCTTG |
|  | BoESSR108 | C04 | GAACTCCACGGAAACCGTTA | CAAGAACCAGACCGACCCTA |
|  | BoESSR122 | C02 | AATCGAAGCTATTCGCCGTA | CATGTGTTGGCTGTTTCAGG |
|  | BoESSR151 | C02 | GCCCACTCCTATTTCCAACA | CTTGGGAAAATCACGGAAGA |
|  | BoESSR206 | C05 | GATGAAGGCTGCTCAAGCTC | CGTCCTCCTCTTCAGCAAAC |
|  | BoESSR207 | C05 | TTGCTGAAGAGGAGGACGAT | CTTCATTTGCACCGGAATCT |
|  | BoESSR208 | C04 | CTTGGGCACTGGATGTTCTT | CACCAAGCAAACTTGGATCA |
|  | BoESSR212 | C07 | AGACAAGACGCCTCGGTTTA | CGTTATGGTAGATACTCAGATGC |
|  | BoESSR216 | C01 | GGTTTCCGCTATGTCCAGAA | CGGAAGAAGACGTTGAGGAG |
|  | BoESSR248 | C04 | GATGTTGGTTTTGGTTTTGG | TGCTTTTGCTTTGATGGTCT |
|  | BoESSR303 | C04 | GAACCCACCTTCCTTCAACA | GCGATTTTCAGGCAGAAGTC |
|  | BoESSR333 | C04 | CCTTGGTCTTCTCCGATGAG | ATGATCGTGAACGTCCCATT |
|  | BoESSR338 | C08 | TGTAGCCGAAAGGGAATGAG | GTGCTTGCATCCAGAAACCT |
|  | BoESSR403 | C08 | TGAGAAGCCTGAGACCACCT | AGATCTGCGCAGCGTTTTAC |
|  | BoESSR409 | C04 | GGGGATTGGTCCTAAAAAGC | ACCAAGGCATCCCTCCTTAG |
|  | BoESSR576 | C06 | CCACGTCGGAGAAGCTAAAG | TGGGAATGATTTTTGGGAGA |
|  | BoESSR581 | C06 | AACGAAGGTGAACCATCAGG | GGCCAGGAGCTGTTACAATG |
|  | BoESSR632 | C01 | CCCTGCAATTGAAAACCAGT | AAACCGTCCAAGGATCATCA |
|  | BoESSR901 | C09 | AGCTAGCAGCGAAGTTCGAG | TTTGTCGGTGGAGAGGATTC |
|  | BoESSR766 | C03 | CGTGTGGAAGACCCTTCACT | TTGGGGAAGTGGTAAGAACG |
|  | BoESSR825 |  | GGACAGCGACACATTGAGTG | GGGAAGAGGTTCCCAAACAT |
|  | BoESSR673 | C03 | GAAAGAGGGAGGGAACGAAG | ACACGGACTCGAATTGGAAC |
|  | BoESSR758 | C07 | ATTTCGAGGTTCTCGCCATT | AACATGTGCCCAATGATGAA |
|  | BoESSR763 | C04 | TCAGGCAGCTAAATTCACACA | GAATGGGCAAAAGACAGCAT |
|  | BoESSR863 | C06 | GAAAGCTAGAGAGTGAGCGAGA | GACGAGGATGGTGACGAATC |
|  | BoESSR903 | C06 | TTCGAAAATGAGACGAGAGG | CCGTATCGGAAGAAGTGGAA |
|  | Na12F03a | C07 | GGCGACATAGATTTGAACCG | TCCACTTTCTCTCTCTTCCCC |
|  | O110B11 | C05 | AAAATGTGAGGCTGTTTGGG | TTTCGCAGCAGTAAACATGG |
|  | BoSF2406 | C07 | ATATGGCCCTGCAAACAGAG | CAGCTTATGGAATGCCCCT |
|  | BoSF2313 | C07 | AAGGAGGATCACGAGGAGGT | CATGGTAGCATCGAAAGCCT |
|  | BoSF2033 | C07 | CCACATTTACGCACTTGCAG | TCCGTATAATGATTTAACAACCCA |
|  | BoSF317 | C05 | CCAACTCCGGTCAATCATCT | GCCCCTTTCTGTGTGACATT |
|  | BoSF2421 | C09 | CACTCAGAGGAGGAGGTTGC | GCCACGTGTAGGCATGTAGA |
|  | BoSF1957 | C04 | TATGGACCACATGCCCCTAT | ACTAGGGGCGGATTCAAAAA |
|  | Na12B09 | C03 | ACGGAAGATCAAACAGCTCC | TGAGCGACCCATTCTTTAGG |
|  | cnu107 | C02 | TGGACGTAACACCCATCTTGAA | AGCTGAGGAAGTGGCTGAGG |
|  | BoSF1131 | C03 | GAAGTTTCACTGCCTCTCGG | CTTCGTTAACCTCGCGAAAG |
|  | BoSF966 | C03 | ATCCCATTGTCGTTATCCCA | CGTCGTCTAGCGATGATGAA |
|  | CB10258 | C01 | ATGATGCCTAGCATGTCC | AAGCTAAAGCGAAAGAAGC |
|  | BoESSR920 | C09 | CATGTACCGGAACTTATTGG | GGAGGAGGGTCCTCTCAATC |
|  | BoESSR041 | C06 | TAGCCGGAACCAAAACTGTC | TGATGCAGAAGAGCAGAGGA |
|  | BoESSR934 | C08 | GTTCGCCAAATCCAAAAGC | ATCTCACTTTCGCCATTGCT |
|  | Ni4D12 | C02 | ACCACCATCCACAGAGTTCC | GCAGGACAGACTGAAAGCG |
|  | cnu149 | C05 | GGAAGCCTCTGTGCGAAAAA | TGCCGACGATTTGATAGAGGA |
|  | BoESSR482 | C02 | GGAAATTCGATAAATCGAGAGC | TCCGGAGGTCCCTTTATTTT |
|  | O112G04a | C08 | CGAACATCTTAGGCCGAATC | GGTTAACCTGCGGGATATTG |
|  | BoESSR492 | C03 | GCGCAGAATCCAGATCATAG | GGCTGGAGTATGAGCGAGAC |
|  | BoESSR510 | C03 | TCAAGTTGGAACGAGAATGTTG | ATTCTTCAATCACGCCTCCTT |
|  | BoESSR523 | C07 | GGACCAAGAGTATCGCAACC | TGATCGCACAAGGAAGTGAG |
|  | BoESSR560 | C03 | GGGATGCAAGCATAACCATT | GACTGCTCGAGTGGTTCCTC |
|  | BoESSR736 | C05 | CCACGGACGGAACAATTTAT | GCTAAAAATGCACCGCTACC |
|  | BoESSR030 | C03 | GTGTGAATGGTGGACAGTCG | TGCTGAGATTGACTCCGTTG |
|  | BoESSR073 | C03 | GGACTGCCAAAAGACTGAGC | ACTCGCACAGGAACCAAAAT |
