# Supplementary material for: Heterosis and combining ability in cytoplasmic male sterile and doubled haploid based Brassica oleracea progenies and prediction of heterosis using microsatellites
Source: PLoS One. 2019 Aug 19;14(8):e0210772. doi: 10.1371/journal.pone.0210772 (PMC6699688; doi:10.1371/journal.pone.0210772)
Supplement: S2 Table — * = significant at 5% probability, ** = significant at 1% probability, *** = significant at 0.1%, **** = significant at 0.01% probability through F test, Rep = Replication, Blk = Block, Trt = Treatment, Rep (Blk)Adj = Rep (Blk) Adjustable Days to 50% CI = Days to 50% curd initiation, Days to 50%CM = Days to 50% curd maturity, PH = Plant height, GPW = Gross plant weight, MCW = marketable curd weight, NCW = net curd weight, LL = leaf length, LW = leaf width, NoL = No of leaves, CL = curd length, CD = curd diameter, CoL = core length, CSI = curd size index, LSI = leaf size index, HI = harvest index, TMY = total marketable yield, R2: coefficient of determination. (DOCX) [file pone.0210772.s004.docx]

**S2 Table.** Estimates of Mean Squares and R^2^ for vegetative and commercial traits in Alpha Lattice Design

| **Source of variation** | **df** | **Days to 50% CI** | **Days to 50% CM** | **PH (cm)** | **GPW (g)** | **MCW (g)** | **NCW (g)** | **LL (cm)** | **LW (cm)** |
| --- | --- | --- | --- | --- | --- | --- | --- | --- | --- |
| Rep | 2 | 39.30**** | 26.16 | 56.54 | 174280.9 | 43664.67 | 17457.74 | 0.79 | 4.81 |
| Rep (Blk)_Adj_ | 27 | 2.25 | 108.25**** | 80.21**** | 472557.5**** | 141692.58**** | 36519.71**** | 121.47**** | 26.45**** |
| Trt | 149 | 5.79**** | 283.87**** | 173.73**** | 1031554.2**** | 243935.75**** | 100214.64**** | 160.75**** | 41.81**** |
| Error | 271 | 0.95 | 5.67 | 10.45 | 19291.1 | 10469.57 | 3294.65 | 4.63 | 2.81 |
| R^2^ |  | 0.79 | 0.96 | 0.91 | 0.96 | 0.93 | 0.94 | 0.95 | 0.91 |

**S2 Table. Continue**

| **Source of variation** | **df** | **NoL** | **CL (cm)** | **CD (cm)** | **CoL (cm)** | **CSI (cm²)** | **LSI (cm²)** | **HI %** | **TMY (t/ha)** |
| --- | --- | --- | --- | --- | --- | --- | --- | --- | --- |
| Rep | 2 | 26.35 | 1.49 | 101.59 | 5658.14 | 10777.72 | 6828.08 | 7.72967 | 69.86 |
| Rep (Blk)_Adj_ | 27 | 11.76**** | 1.76**** | 41.71 | 938.56 | 4369.41 | 222739.95**** | 130.65**** | 226.71**** |
| Trt | 149 | 27.59**** | 2.15**** | 47.54 | 1419.06 | 5052.84 | 312809.74**** | 219.61**** | 390.29**** |
| Error | 271 | 3.97 | 0.62 | 43.15 | 1410.16 | 3930.53 | 8735.85 | 28.65 | 16.75 |
| R^2^ |  | 0.81 | 0.68 | 0.42 | 0.39 | 0.45 | 0.95 | 0.82 | 0.93 |

*****= significant at 5% probability, ******= significant at 1% probability, *******= significant at 0.1%, ********= significant at 0.01% probability through F test, Rep = Replication, Blk = Block, Trt = Treatment, Rep (Blk)_Adj_= Rep (Blk) Adjustable Days to 50% CI= Days to 50% curd initiation, Days to 50%CM= Days to 50% curd maturity, PH= Plant height, GPW= Gross plant weight, MCW= marketable curd weight, NCW = net curd weight, LL= leaf length, LW= leaf width, NoL= No of leaves, CL= curd length, CD= curd diameter, CoL= core length, CSI= curd size index, LSI= leaf size index, HI= harvest index, TMY= total marketable yield, R^2^: coefficient of determination
