# Supplementary material for: Heterosis and combining ability in cytoplasmic male sterile and doubled haploid based Brassica oleracea progenies and prediction of heterosis using microsatellites
Source: PLoS One. 2019 Aug 19;14(8):e0210772. doi: 10.1371/journal.pone.0210772 (PMC6699688; doi:10.1371/journal.pone.0210772)
Supplement: S3 Table — * = significant at 5% probability, ** = significant at 1% probability, *** = significant at 0.1%, **** = significant at 0.01% probability through F test, CD = critical difference, Days to 50% CI = Days to 50% curd initiation, Days to 50%CM = Days to 50% curd maturity, PH = Plant height, GPW = Gross plant weight, MCW = marketable curd weight, NCW = net curd weight, LL = leaf length, LW = leaf width, NoL = No of leaves, CL = curd length, CD = curd diameter, CoL = core length, CSI = curd size index, LSI = leaf size index, HI = harvest index, TMY = total marketable yield. (DOCX) [file pone.0210772.s005.docx]

**S3 Table.** Estimates of SCA effects of 120 test cross progenies for yield and horticultural traits

|  | **Days to 50% CI** | **Days to 50% CM** | **PH (cm)** | **GPW (g)** | **MCW (g)** | **NCW (g)** | **LL (cm)** | **LW (cm)** | **NoL** | **CL (cm)** | **CD (cm)** | **CoL (cm)** | **CSI (cm²)** | **LSI (cm²)** | **HI %** | **TMY (t/ha)** |
| --- | --- | --- | --- | --- | --- | --- | --- | --- | --- | --- | --- | --- | --- | --- | --- | --- |
| Ogu122-5A*DH-18-8-1 | -1.08 | -1.35 | 3.29 | 167.38* | -84.64 | -49.11 | -0.78 | 2.31** | -0.93 | -2.81*** | -1.23** | 0.08 | -44.62*** | 75.65 | -9.12** | -3.39 |
| Ogu122-5A*DH-18-8-3 | 0.00 | -0.33 | -2.13 | -22.67 | -193.98** | -62.26 | 9.26*** | 3.84*** | 0.70 | 0.54 | -0.38 | -0.73*** | -0.02 | 440.56*** | -8.95** | -7.76** |
| Ogu122-5A*DH-53-1 | -0.10 | 1.30 | -6.35** | -678.08*** | -374.71*** | -169.67*** | -11.01*** | -2.10* | -1.42 | 0.44 | -0.64 | -0.19 | -0.39 | -346.30*** | -0.96 | -14.99*** |
| Ogu122-5A*DH-53-6 | -0.16 | 1.67 | 4.92* | 409.99*** | 189.07** | 100.24** | 8.96*** | 1.01 | 2.37* | 1.13* | 1.44** | 0.62** | 28.74*** | 250.35*** | -1.52 | 7.56** |
| Ogu122-5A*DH-53-9 | 0.39 | 0.47 | -6.54*** | -966.86*** | -266.78*** | -100.01** | -5.97*** | -2.83*** | -2.25 | -0.59 | -0.53 | -0.40* | -12.63 | -286.24*** | 17.39*** | -10.67*** |
| Ogu122-5A*DH-53-10 | 0.95 | -1.75 | 6.81*** | 1090.24*** | 731.04*** | 280.79*** | -0.47 | -2.24** | 1.53 | 1.30** | 1.35** | 0.61** | 28.92*** | -134.02* | 3.16 | 29.24*** |
| Ogu115-33A*DH-18-8-1 | -0.64 | 0.87 | -6.17** | -455.13*** | -99.31 | -75.49* | -12.67*** | -1.32 | -3.21** | -0.89 | -2.42*** | -0.96*** | -31.71*** | -363.46*** | 3.69 | -3.97 |
| Ogu115-33A*DH-18-8-3 | 1.45* | 2.56* | -2.88 | -321.17*** | 23.69 | 33.36 | 8.01*** | 1.21 | -0.58 | 0.62 | 1.13* | 0.61** | 16.38* | 247.61*** | 8.58** | 0.95 |
| Ogu115-33A*DH-53-1 | 1.01 | 1.52 | 1.35 | 61.76 | -130.38* | 14.27 | -12.60*** | -5.03*** | -0.69 | -0.01 | 0.11 | 0.38* | 1.36 | -538.90*** | -5.38 | -5.22* |
| Ogu115-33A*DH-53-6 | -1.05 | -8.11*** | 7.58*** | 176.49* | 68.74 | 55.19 | 7.14*** | 3.39*** | 3.09** | 0.35 | 0.89* | 0.45* | 12.65 | 345.14*** | -0.89 | 2.75 |
| Ogu115-33A*DH-53-9 | 0.16 | 2.36 | 1.02 | 692.97*** | 201.89*** | 108.61** | 6.00*** | 0.71 | -2.53* | 0.74 | 0.65 | -0.39* | 14.55* | 170.06** | -5.81 | 8.08*** |
| Ogu115-33A*DH-53-10 | -0.94 | 0.81 | -0.90 | -154.93 | -64.63 | -135.93*** | 4.11** | 1.04 | 3.92*** | -0.81 | -0.37 | -0.09 | -13.22 | 139.55* | -0.20 | -2.59 |
| Ogu118-6A*DH-18-8-1 | -0.08 | 4.09** | -6.83*** | -207.63* | 30.97 | 68.78* | -8.56*** | -4.03*** | -2.38* | -0.02 | 0.13 | 0.13 | 2.27 | -370.99*** | 5.02 | 1.24 |
| Ogu118-6A*DH-18-8-3 | -0.33 | 3.78** | -7.65*** | -711.01*** | -303.03*** | -352.70*** | -8.25*** | -0.77 | -2.41* | 0.20 | -0.96* | 0.03 | -8.18 | -257.5*** | 1.13 | -12.12*** |
| Ogu118-6A*DH-53-1 | -0.43 | 2.41 | 0.65 | -42.74 | 27.90 | -14.45 | 0.04 | 1.33 | 0.81 | 0.23 | 0.66 | -0.22 | 9.49 | 70.98 | 2.83 | 1.12 |
| Ogu118-6A*DH-53-6 | -0.83 | -6.89*** | -0.42 | -180.34* | -86.98 | -75.87* | 2.35 | 1.01 | 1.26 | -0.41 | 0.40 | 0.37 | -1.27 | 88.27 | -0.36 | -3.48 |
| Ogu118-6A*DH-53-9 | 0.72 | -5.42*** | 11.18*** | 123.47 | -13.83 | 41.55 | 10.71*** | 1.30 | 0.31 | -0.29 | -0.24 | -0.36 | -5.90 | 330.96*** | -4.49 | -0.55 |
| Ogu118-6A*DH-53-10 | 0.95 | 2.03 | 3.07 | 1018.24*** | 344.98*** | 332.68*** | 3.72** | 1.16 | 2.42* | 0.29 | 0.01 | 0.05 | 3.58 | 138.28* | -4.14 | 13.80*** |
| Ogu307-33A*DH-18-8-1 | 0.81 | -4.74*** | 5.27** | 834.10*** | -40.64 | 165.84*** | 3.89** | 1.03 | 4.29*** | -0.13 | 1.72*** | 0.26 | 13.49 | 116.75* | -16.69*** | -1.63 |
| Ogu307-33A*DH-18-8-3 | -5.44*** | -3.39** | 10.93*** | 1172.05*** | 584.02*** | 144.36*** | 8.46*** | 2.69** | 3.92*** | 0.85 | 0.77 | 0.68*** | 16.12* | 354.18*** | -0.84 | 23.36*** |
| Ogu307-33A*DH-53-1 | 1.79** | 6.24*** | -7.11*** | -809.02*** | -438.04*** | -166.73*** | -10.94*** | -3.05*** | -2.53* | 0.29 | -1.45** | -0.46* | -9.29 | -428.05*** | -3.33 | -17.52*** |
| Ogu307-33A*DH-53-6 | 1.06 | 9.28*** | -11.28*** | -1193.95*** | -416.26*** | -228.81*** | -16.04*** | -4.57*** | -2.08 | -0.85 | -0.82 | -*0.50 | -16.54* | -596.24*** | 10.60*** | -16.65*** |
| Ogu307-33A*DH-53-9 | 1.61* | -2.26 | 3.12 | -197.14* | 72.89 | -18.73 | 7.93*** | 1.42 | 0.31 | 0.23 | -0.38 | -0.37 | -0.59 | 262.31*** | 5.31 | 2.92 |
| Ogu307-33A*DH-53-10 | 0.17 | -5.14*** | -0.93 | 193.96* | 238.04*** | 104.07** | 6.70*** | 2.48** | -3.9***1 | -0.38 | 0.17 | 0.39* | -3.19 | 291.06*** | 4.95 | 9.52*** |
| Ogu309-2A*DH-18-8-1 | 0.81 | 0.37 | -1.72 | -354.13*** | -194.70** | -173.16*** | -1.10 | 1.07 | 0.46 | -0.29 | -0.54 | -0.17 | -8.40 | 33.62 | 1.14 | -7.79** |
| Ogu309-2A*DH-18-8-3 | 0.89 | 1.06 | -2.96 | 12.49 | -3.03 | 55.36 | -4.40*** | -1.81* | 1.76 | -0.81 | -1.17* | -0.79*** | -20.19** | -180.50*** | 0.17 | -0.12 |
| Ogu309-2A*DH-53-1 | 0.79 | -2.64* | -7.50*** | -487.24*** | -297.10*** | -159.39*** | -5.14*** | -3.68*** | -0.36 | 0.06 | 0.28 | -0.89*** | 3.37 | -267.21*** | -0.68 | -11.88*** |
| Ogu309-2A*DH-53-6 | -0.61 | 0.39 | 5.80** | 552.49*** | 527.68*** | 337.52*** | 4.70*** | 3.74*** | 0.76 | 0.36 | 0.36 | 0.48* | 7.96 | 271.62*** | 9.07** | 21.11*** |
| Ogu309-2A*DH-53-9 | -2.72*** | 0.19 | 3.07 | 200.64* | -90.17 | -60.73 | 4.66*** | 2.23** | -0.86 | 0.25 | -0.15 | 0.52** | 0.55 | 193.94*** | -10.66*** | -3.61 |
| Ogu309-2A*DH-53-10 | 0.84 | 0.64 | 3.32 | 75.74 | 57.32 | 0.41 | 1.27 | -1.55 | -1.74 | 0.42 | 1.23** | 0.85*** | 16.71* | -51.46 | 0.96 | 2.29 |
| Ogu33A*DH-18-8-1 | -0.30 | -3.29* | 7.54*** | 274.54*** | -89.03 | -62.94 | 8.39*** | 3.11*** | 2.79* | -0.40 | 0.04 | 0.13 | -4.73 | 334.36*** | -9.58** | -3.56 |
| Ogu33A*DH-18-8-3 | 0.78 | 5.39*** | 1.46 | 862.16*** | -14.70 | 56.58 | 6.23*** | 4.17*** | 3.42** | -0.39 | -1.12* | -0.15 | -17.00* | 341.59*** | -13.95*** | -0.59 |
| Ogu33A*DH-53-1 | -1.65** | -2.64* | -8.61*** | 29.09 | 906.90*** | 432.16*** | -5.01*** | -4.17*** | 3.64** | 0.45 | 0.54 | 0.73*** | 12.40 | -313.54*** | 33.46*** | 36.28*** |
| Ogu33A*DH-53-6 | -0.72 | -4.94*** | -1.45 | -122.51 | -155.32** | -191.59*** | -7.67*** | -3.95*** | -2.91* | -0.09 | 0.29 | -0.41* | 1.34 | -339.70*** | -3.90 | -6.21** |
| Ogu33A*DH-53-9 | 0.83 | -0.48 | 3.79* | -102.69 | -182.50** | 7.49 | 6.66*** | 2.27** | -1.19 | 0.39 | 0.44 | 0.15 | 9.08 | 249.86*** | -6.74* | -7.30** |
| Ogu33A*DH-53-10 | 1.06 | 5.97*** | -2.73 | -940.59*** | -465.35*** | -241.71*** | -8.60*** | -1.44 | -5.74*** | 0.04 | -0.20 | -0.45* | -1.09 | -272.57*** | 0.72 | -18.61*** |
| OguKt-2-6A*DH-18-8-1 | 0.14 | -0.07 | 5.59** | 102.43 | 140.86* | 10.28 | 0.09 | -1.02 | -2.60* | 0.52 | 0.48 | -0.40* | 11.35 | -72.03 | 2.10 | 5.63* |
| OguKt-2-6A*DH-18-8-3 | -0.11 | 3.28* | 3.21 | -193.62* | -95.14 | -39.53 | -2.84* | -3.56*** | 0.37 | -0.50 | 0.00 | 0.58** | -8.54 | -275.23*** | -0.28 | -3.81 |
| OguKt-2-6A*DH-53-1 | 0.46 | -0.76 | -3.26 | 569.98*** | 221.79*** | 90.72** | 4.92*** | 2.47** | -2.75* | -0.03 | 0.69 | 0.41* | 6.77 | 326.37*** | -0.24 | 8.87*** |
| OguKt-2-6A*DH-53-6 | 0.39 | 1.94 | -9.43*** | -337.62*** | -13.76 | -38.37 | 0.40 | -1.05 | -0.63 | -0.34 | -1.37** | -0.05 | -16.62* | -60.73 | 6.37* | -0.55 |
| OguKt-2-6A*DH-53-9 | -0.73 | -6.92*** | 6.47*** | 408.53*** | 76.39 | 100.72** | -3.27** | 5.27*** | 0.08 | 0.45 | 1.13* | -0.32 | 16.71* | 186.88*** | -5.43 | 3.06 |
| OguKt-2-6A*DH-53-10 | -0.16 | 2.53* | -2.58 | -549.70*** | -330.13*** | -123.82*** | 0.70 | -2.10** | 5.53*** | -0.10 | -0.92* | -0.22 | -9.68 | -105.26 | -2.52 | -13.21*** |
| Ogu1A*DH-18-8-1 | 0.25 | 2.37 | -1.46 | -7.46 | -155.03** | -112.77*** | -3.62** | -2.06* | -0.71 | 0.95* | -0.33 | 0.07 | 8.19 | -170.48** | -8.98** | -6.20** |
| Ogu1A*DH-18-8-3 | -1.66** | -11.61*** | -4.61* | -163.51* | -135.03* | -111.26*** | -1.91 | -1.83* | -0.08 | -1.17* | -0.48 | 0.30 | -21.62** | -151.56** | -2.92 | -5.40* |
| Ogu1A*DH-53-1 | 0.90 | 1.36 | 8.52*** | -79.57 | -103.77 | -167.01*** | 1.55 | 2.43** | -3.53** | 1.03* | 0.72 | -0.46* | 21.91** | 160.17** | -2.06 | -4.15 |
| Ogu1A*DH-53-6 | 0.84 | 2.72* | 5.22** | -95.84 | 107.68 | 168.58*** | 8.79*** | 4.18*** | 3.59** | -0.46 | 0.42 | -0.52** | -2.83 | 412.28*** | 7.78* | 4.31 |
| Ogu1A*DH-53-9 | -0.61 | 3.52** | 1.42 | 1022.31*** | 525.17*** | 415.99*** | 2.59* | 1.07 | 2.31* | 0.91 | 1.25** | 0.91*** | 24.12** | 94.26 | -1.63 | 21.01*** |
| Ogu1A*DH-53-10 | 0.29 | 1.64 | -9.09*** | -675.92*** | -239.02*** | -193.54*** | -7.40*** | -3.80*** | -1.58 | -1.26** | -1.58*** | -0.30 | -29.77*** | -344.68*** | 7.82** | -9.56*** |
| Ogu13-85-6A*DH-18-8-1 | -1.08 | -1.79 | -3.40 | -15.29 | 101.69 | -174.16*** | 0.85 | 0.87 | 4.84*** | 0.43 | -0.02 | -0.34 | 4.41 | 49.78 | 1.62 | 4.07 |
| Ogu13-85-6A*DH-18-8-3 | 0.00 | -1.11 | 11.18*** | 44.66 | 160.02** | 221.36*** | -2.98* | -0.07 | -1.52 | 0.83 | 2.17*** | 1.09*** | 28.08*** | -97.12 | 3.31 | 6.40** |
| Ogu13-85-6A*DH-53-1 | 0.57 | -9.81*** | 13.61*** | 267.59** | 94.96 | 131.61*** | 13.58*** | 3.46*** | 3.36** | -0.65 | -0.85 | 0.01 | -14.92* | 449.52*** | -2.69 | 3.80 |
| Ogu13-85-6A*DH-53-6 | 0.17 | 9.56*** | -3.39 | 748.66*** | 175.74** | 204.86*** | -3.71** | -0.96 | -3.86** | 0.07 | -0.62 | -0.06 | -4.72 | -125.25* | -8.31** | 7.03** |
| Ogu13-85-6A*DH-53-9 | 0.72 | 6.36*** | -12.79*** | -1253.86*** | -440.11*** | -297.06*** | -11.82*** | -6.43*** | -5.47*** | -0.86 | -1.46** | -0.91*** | -21.93** | -469.79*** | 16.39*** | -17.60*** |
| Ogu13-85-6A*DH-53-10 | -0.38 | -3.19* | -5.21** | 208.24* | -92.29 | -86.59** | 4.09** | 3.13*** | 2.64* | 0.18 | 0.78 | 0.22 | 9.09 | 192.86*** | -10.32*** | -3.69 |
| Ogu1-6A*DH-18-8-1 | -0.25 | 0.48 | 0.93 | -328.18*** | -103.76 | -12.66 | -1.30 | -0.21 | -1.66 | 0.55 | 0.12 | 0.32 | 8.50 | -15.49 | 5.13 | -4.15 |
| Ogu1-6A*DH-18-8-3 | 1.50* | 3.50** | -8.38*** | -474.90*** | -337.09*** | -257.48*** | -3.39** | -3.19*** | 0.31 | -0.03 | -2.24*** | -0.91*** | -23.32** | -213.36*** | -3.85 | -13.48*** |
| Ogu1-6A*DH-53-1 | 0.07 | 2.47 | -1.26 | 849.37*** | 715.84*** | 361.77*** | 6.07*** | 1.75* | 1.53 | 0.29 | 1.40** | 1.06*** | 17.53* | 217.07*** | 9.54** | 28.63*** |
| Ogu1-6A*DH-53-6 | 0.34 | 1.50 | 5.11** | -275.90*** | -216.04*** | -103.31** | 5.04*** | 1.42 | -0.36 | 0.23 | 0.63 | 0.05 | 9.46 | 167.71** | -2.90 | -8.64*** |
| Ogu1-6A*DH-53-9 | 0.55 | -0.03 | 0.28 | 189.25* | 65.78 | 44.77 | -2.00 | -1.45 | -3.31** | -0.30 | 0.22 | -0.46* | -1.95 | -116.77* | -2.04 | 2.63 |
| Ogu1-6A*DH-53-10 | -2.21*** | -7.92*** | 3.33 | 40.35 | -124.74* | -33.09 | -4.42*** | 1.68* | 3.48** | -0.74 | -0.13 | -0.05 | -10.21 | -39.15 | -5.88 | -4.99* |
| Ogu2A*DH-18-8-1 | -0.58 | 0.93 | 9.28*** | -36.18 | -21.87 | -47.22 | 1.36 | -1.23 | -0.32 | 0.25 | 0.10 | -1.03*** | 3.50 | -16.21 | -0.89 | -0.87 |
| Ogu2A*DH-18-8-3 | 0.50 | -10.06*** | 2.37 | 44.77 | -16.87 | -85.37* | -5.50*** | 0.03 | 3.64** | 0.22 | 0.06 | 0.17 | 0.79 | -124.79* | -2.28 | -0.67 |
| Ogu2A*DH-53-1 | -0.93 | 2.58* | -7.90*** | -497.96*** | -160.27** | -119.45*** | 0.76 | -3.60*** | -3.14** | -0.94* | -0.80 | -0.10 | -19.42** | -160.40** | 6.51 | -6.41** |
| Ogu2A*DH-53-6 | 1.00 | 1.61 | 2.03 | -28.23 | 25.52 | 3.13 | 5.04*** | 4.94*** | -5.02*** | 1.10* | 2.09*** | 0.93*** | 34.85*** | 350.11*** | 2.12 | 1.02 |
| Ogu2A*DH-53-9 | -0.45 | 1.41 | -6.60*** | 372.59*** | 303.00*** | 248.22*** | 2.43 | 1.37 | 5.36*** | 0.49 | 0.69 | 0.90*** | 12.08 | 108.37* | 4.21 | 12.12*** |
| Ogu2A*DH-53-10 | 0.45 | 3.53** | 0.82 | 145.02 | -129.52* | 0.68 | -4.09** | -1.51 | -0.52 | -1.12* | -2.14*** | -0.88*** | -31.80*** | -157.08** | -9.67** | -5.18* |
| OguKt-9-2A*DH-18-8-1 | 0.98 | -1.24 | -8.96*** | 102.82 | 81.30 | 94.12** | -3.43** | -1.27 | -1.27 | -0.49 | -1.84*** | -0.46* | -23.86** | -136.33* | 1.17 | 3.25 |
| OguKt-9-2A*DH-18-8-3 | 0.39 | -1.22 | 3.23 | 451.44*** | 234.63*** | 235.97*** | 4.21*** | 0.92 | 0.03 | 0.31 | 1.91*** | 0.47* | 19.16** | 130.58* | 0.06 | 9.39*** |
| OguKt-9-2A*DH-53-1 | -0.04 | 0.41 | 0.12 | -286.96*** | -152.43* | -101.45** | -0.16 | 1.15 | 0.92 | -0.54 | -1.29** | -0.38* | -19.27** | 55.78 | 0.71 | -6.10* |
| OguKt-9-2A*DH-53-6 | -1.11 | 1.11 | 8.65*** | 255.10** | 198.35*** | 147.80*** | 9.25*** | 4.66*** | 2.37* | 0.52 | -0.37 | -0.27 | 1.35 | 459.94*** | 3.55 | 7.93*** |
| OguKt-9-2A*DH-53-9 | -0.56 | 0.24 | -6.81*** | -207.75* | -150.50* | -156.78*** | -5.02*** | -5.18*** | -0.92 | -0.05 | 0.54 | 0.26 | 10.33 | -366.34*** | -3.30 | -6.02* |
| OguKt-9-2A*DH-53-10 | 0.34 | 0.69 | 3.77 | -314.65*** | -211.35*** | -219.65*** | -4.85*** | -0.29 | -1.13 | 0.24 | 1.05* | 0.39* | 12.29 | -143.62** | -2.20 | -8.45*** |
| Ogu22-1A*DH-18-8-1 | -0.14 | 2.15 | -4.08* | 404.04*** | 176.58** | 78.95* | 5.00*** | 3.94*** | -2.27 | 0.01 | 0.21 | -0.26 | -5.92 | 275.61*** | -4.13 | 7.06** |
| Ogu22-1A*DH-18-8-3 | 0.95 | 4.50*** | -4.13* | -360.67*** | -9.09 | -30.87 | 6.24*** | 2.47** | 1.70 | -2.09*** | 0.66 | 0.34 | 17.63* | 221.01*** | 9.24** | -0.36 |
| Ogu22-1A*DH-53-1 | -0.15 | -3.87** | -0.30 | -544.41*** | -327.16*** | -157.28*** | 8.03*** | 5.43*** | -1.42 | 0.77 | -0.78 | 0.01 | -5.54 | 453.02*** | -3.98 | -13.09*** |
| Ogu22-1A*DH-53-6 | 0.11 | 5.50*** | 2.33 | 1175.32*** | 295.29*** | 391.30*** | 0.41 | -1.95* | 3.70** | 0.58 | 1.67*** | 0.47* | 15.29* | -104.13 | -11.22*** | 11.81*** |
| Ogu22-1A*DH-53-9 | 0.33 | 5.30*** | 3.37 | -394.86*** | 34.11 | -88.95** | -9.23*** | -4.76*** | -2.25 | -0.24 | -1.33** | -0.74*** | -21.65** | -410.43*** | 12.81*** | 1.36 |
| Ogu22-1A*DH-53-10 | -1.10 | -13.58*** | 2.82 | -279.42*** | -169.74** | -193.15*** | -10.45*** | -5.14*** | 0.53 | 0.96* | -0.41 | 0.18 | 0.19 | -435.08*** | -2.72 | -6.79** |
| Ogu122-1A*DH-18-8-1 | 0.42 | -1.91 | -1.77 | 229.04** | 142.08* | 69.95* | -2.93* | -0.88 | -1.82 | 0.03 | -0.57 | 0.30 | -6.00 | -116.67* | -0.82 | 5.68* |
| Ogu122-1A*DH-18-8-3 | 0.50 | -1.22 | 13.82*** | 525.99*** | -129.59* | 60.13 | 5.91*** | 4.18*** | -0.52 | -0.14 | 0.91* | 0.13 | 3.84 | 359.43*** | -17.57*** | -5.18* |
| Ogu122-1A*DH-53-1 | 0.07 | 0.41 | 4.88* | 393.92*** | 234.34*** | 21.05 | 1.67 | 0.65 | 3.03** | 0.15 | 0.39 | 0.28 | 5.05 | 75.46 | 1.37 | 9.37*** |
| Ogu122-1A*DH-53-6 | -1.00 | 0.44 | -12.59*** | -3.01 | -28.21 | -133.03*** | -7.53*** | -4.68*** | 3.81** | -0.74 | -0.69 | -0.88*** | -15.17* | -390.35*** | -2.55 | -1.13 |
| Ogu122-1A*DH-53-9 | 0.55 | 0.91 | -12.25*** | -851.53*** | -379.06*** | -280.95*** | -4.46*** | -1.02 | -2.14 | -0.99* | -2.00*** | -0.22 | -29.21*** | -174.69** | 2.48 | -15.16*** |
| Ogu122-1A*DH-53-10 | -0.55 | 1.36 | 7.90*** | -294.42*** | 160.43** | 262.85*** | 7.35*** | 1.74* | -2.36* | 1.69*** | 1.96*** | 0.38* | 41.50*** | 246.82*** | 17.08*** | 6.42** |
| Ogu126-1A*DH-18-8-1 | -1.58* | 1.09 | -2.28 | -702.90*** | -376.92*** | -242.66*** | -7.43*** | -5.08*** | -2.38* | 0.08 | 1.29** | 0.87*** | 13.86 | -403.64*** | -0.18 | -15.08*** |
| Ogu126-1A*DH-18-8-3 | 0.50 | 4.78*** | 1.90 | 564.05*** | 676.08*** | 209.86*** | 3.71*** | -0.02 | -3.08** | -0.33 | -0.96* | -0.37 | -15.03* | 59.57 | 12.52*** | 27.04*** |
| Ogu126-1A*DH-53-1 | 1.07 | 4.41*** | 3.80* | 800.31*** | -23.32 | 122.44*** | 4.77*** | 3.31*** | 2.47* | 0.75 | 0.97* | 0.12 | 19.58** | 313.65*** | -14.40*** | -0.93 |
| Ogu126-1A*DH-53-6 | 0.00 | -0.56 | 4.36* | -274.95*** | -118.54* | 32.36 | -2.02 | -3.38*** | -0.08 | 0.24 | -0.55 | 0.03 | -2.71 | -241.57*** | 1.02 | -4.74* |
| Ogu126-1A*DH-53-9 | -0.45 | -8.09*** | 0.57 | 39.86 | 4.94 | 15.44 | -4.76*** | 0.28 | 3.97*** | -0.69 | -0.79 | 0.25 | -16.10* | -125.39* | -2.57 | 0.20 |
| Ogu126-1A*DH-53-10 | 0.45 | -1.64 | -8.35*** | -426.37*** | -162.24** | -137.43*** | 5.72*** | 4.90*** | -0.91 | -0.05 | 0.04 | -0.91*** | 0.41 | 397.38*** | 3.61 | -6.49** |
| Ogu12A*DH-18-8-1 | 0.75 | -0.79 | 6.70*** | 223.76** | 140.41* | 134.62*** | 10.26*** | 6.13*** | 1.23 | 0.05 | -0.39 | 0.30 | -3.43 | 531.19*** | 0.32 | 5.62* |
| Ogu12A*DH-18-8-3 | -0.83 | 1.89 | -2.98 | 9.05 | 214.41*** | 238.80*** | 0.20 | -4.08*** | -2.80* | 1.16* | 1.85*** | 0.70*** | 30.28*** | -199.63*** | 12.71*** | 8.58*** |
| Ogu12A*DH-53-1 | 1.07 | 2.52 | -6.02** | 78.65 | -89.99 | -33.28 | -0.34 | -1.02 | -2.25 | -0.32 | 0.84 | 0.45* | 3.12 | -61.72 | -7.10* | -3.60 |
| Ogu12A*DH-53-6 | -0.33 | 1.56 | 5.58** | -148.29 | -47.54 | -174.37*** | -4.80*** | -1.54 | -0.47 | 0.49 | -0.49 | -0.87*** | 1.30 | -164.79** | 2.09 | -1.90 |
| Ogu12A*DH-53-9 | -0.78 | -1.31 | 0.62 | -90.14 | -34.72 | -56.28 | 0.83 | 2.59** | 1.92 | -0.02 | -0.03 | 0.71*** | -0.55 | 121.03* | 0.40 | -1.39 |
| Ogu12A*DH-53-10 | 0.12 | -3.86** | -3.90* | -73.04 | -182.57** | -109.48** | -6.16*** | -2.09* | 2.37* | -1.37** | -1.77*** | -1.30*** | -30.71*** | -226.08*** | -8.42** | -7.30** |
| Ogu119-1A*DH-18-8-1 | -0.30 | 0.48 | 1.48 | 46.54 | 61.63 | -1.72 | 9.35*** | 2.48** | 1.62 | 2.09*** | 1.38** | 0.72*** | 42.09*** | 330.64*** | 1.25 | 2.47 |
| Ogu119-1A*DH-18-8-3 | -0.89 | -6.17*** | -3.06 | -591.51*** | -264.37*** | -146.20*** | -2.98* | 1.24 | -0.74 | -0.68 | -1.16* | -0.28 | -22.08** | -18.12 | 2.96 | -10.57*** |
| Ogu119-1A*DH-53-1 | 1.35* | -4.20** | -2.43 | -141.91 | -345.43*** | -189.62*** | -11.52*** | -4.77*** | -2.53* | -2.36*** | -1.50** | -1.00*** | -42.59*** | -449.70*** | -14.31*** | -13.82*** |
| Ogu119-1A*DH-53-6 | 0.61 | 0.83 | -0.27 | 32.83 | 71.35 | -6.37 | 1.69 | 1.51 | 2.59* | 0.77 | 0.17 | 0.43* | 11.40 | 106.64* | 2.85 | 2.85 |
| Ogu119-1A*DH-53-9 | -0.84 | 15.63*** | 4.77* | 469.31*** | 184.50** | 121.72*** | 0.02 | -0.53 | 2.64* | -0.72 | -0.09 | -0.50* | -11.73 | -37.57 | -3.38 | 7.38** |
| Ogu119-1A*DH-53-10 | 0.06 | -6.58*** | -0.48 | 184.74* | 292.32*** | 222.18*** | 3.43** | 0.06 | -3.58** | 0.90 | 1.21** | 0.62** | 22.91** | 68.11 | 10.63*** | 11.69*** |
| Ogu34-1A*DH-18-8-1 | 2.09** | -1.63 | -7.29*** | 314.93*** | 127.91* | 165.12*** | 0.38 | -1.61* | 2.68* | 1.24** | 1.79*** | 0.46* | 32.82*** | -97.03 | -1.77 | 5.12* |
| Ogu34-1A*DH-18-8-3 | 0.50 | -0.61 | -7.67*** | -709.79*** | -374.76*** | -229.70*** | -9.65*** | -3.15*** | -2.69* | -0.38 | -1.35** | -0.82*** | -18.70* | -397.53*** | -0.54 | -14.99*** |
| Ogu34-1A*DH-53-1 | -3.26*** | 3.02* | 8.99*** | 355.48*** | 225.84*** | 109.55** | 5.98*** | 2.55** | 4.53*** | -0.25 | -0.32 | -0.49* | -6.56 | 282.31*** | 3.19 | 9.03*** |
| Ogu34-1A*DH-53-6 | 1.67** | -2.28 | -3.75 | -566.12*** | -325.04*** | -237.20*** | -9.51*** | -5.41*** | -6.02*** | -0.97* | -1.54** | -0.16 | -24.15** | -468.32*** | -2.25 | -13.00*** |
| Ogu34-1A*DH-53-9 | 0.89 | -3.81** | 8.96*** | 453.03*** | 237.44*** | 55.88 | 2.68* | 2.35** | 3.36** | 0.51 | 1.70*** | 1.11*** | 21.43** | 155.93** | -0.59 | 9.50*** |
| Ogu34-1A*DH-53-10 | -1.88** | 5.31*** | 0.77 | 152.46 | 108.59 | 136.35*** | 10.12*** | 5.28*** | -1.86 | -0.15 | -0.28 | -0.10 | -4.84 | 524.64*** | 1.96 | 4.34 |
| Ogu125-8A*DH-18-8-1 | 0.09 | -4.91*** | 6.79*** | -296.96*** | 181.80** | 186.34*** | 4.03** | 0.87 | 3.90*** | -0.12 | 1.40** | 0.79*** | 11.59 | 120.17* | 17.53*** | 7.27** |
| Ogu125-8A*DH-18-8-3 | 0.50 | 9.44*** | 1.31 | -12.34 | 89.13 | 159.86*** | -0.46 | 3.03*** | 1.87 | 1.13* | 0.83 | -0.43* | 21.30** | 123.25* | 3.34 | 3.57 |
| Ogu125-8A*DH-53-1 | -1.93** | -3.92** | 1.00 | 309.26*** | 78.07 | -35.89 | 9.03*** | 4.26*** | -3.58** | -0.07 | -0.11 | 0.59** | -1.59 | 489.86*** | -3.33 | 3.12 |
| Ogu125-8A*DH-53-6 | -0.33 | -12.89*** | -5.30** | 107.66 | -62.82 | -155.98*** | -0.36 | 1.20 | -0.80 | -1.36** | -1.74*** | -0.59** | -32.46*** | 29.98 | -5.53 | -2.51 |
| Ogu125-8A*DH-53-9 | 0.55 | 0.58 | 1.61 | 58.47 | -125.33* | -17.89 | -7.14*** | -4.44*** | 1.92 | 0.71 | 0.53 | 0.05 | 13.92 | -393.00*** | -8.47** | -5.01* |
| Ogu125-8A*DH-53-10 | 1.12 | 11.69*** | -5.41** | -166.09* | -160.85** | -136.43*** | -5.09*** | -4.91*** | -3.30** | -0.30 | -0.91* | -0.41* | -12.76 | -370.27*** | -3.54 | -6.43** |
| Ogu33-1A*DH-18-8-1 | -0.30 | 8.87*** | -2.92 | -295.74*** | -19.31 | -22.11 | -1.80 | -3.11*** | -2.27 | -1.08* | -1.32** | -0.81*** | -23.39** | -105.41 | 13.20*** | -0.77 |
| Ogu33-1A*DH-18-8-3 | 0.78 | -4.44*** | -2.97 | -125.45 | -105.31 | -100.26** | -9.90*** | -5.28*** | -3.30** | 0.65 | -0.47 | -0.63** | 1.09 | -362.43*** | -2.85 | -4.21 |
| Ogu33-1A*DH-53-1 | -0.65 | -0.81 | 7.83*** | -147.52 | -63.04 | 30.66 | 0.33 | -1.38 | 3.92*** | 0.72 | 1.13* | 0.15 | 18.99** | -328.36*** | 0.86 | -2.52 |
| Ogu33-1A*DH-53-6 | -0.05 | -2.44 | -3.68 | -231.79** | -188.93** | -96.09** | -2.13 | 0.43 | -1.30 | -0.62 | -0.15 | 0.46* | -7.84 | 9.04 | -6.03* | -7.56** |
| Ogu33-1A*DH-53-9 | -0.17 | -8.64*** | -5.24** | 34.36 | -23.11 | -83.01* | 9.16*** | 5.76*** | -1.25 | 0.06 | -0.14 | -0.20 | -0.53 | 506.62*** | -3.88 | -0.92 |
| Ogu33-1A*DH-53-10 | 0.40 | 7.47*** | 6.98*** | 766.13*** | 399.71*** | 270.79*** | 4.34*** | 3.58*** | 4.20*** | 0.27 | 0.94* | 1.03*** | 11.68 | 280.55*** | -1.29 | 15.99*** |
| CD 95% SCA | 1.25 | 2.53 | 3.78 | 162.08 | 115.92 | 65.30 | 2.46 | 1.59 | 2.29 | 0.91 | 0.89 | 0.38 | 14.30 | 105.95 | 5.92 | 4.64 |

*****= significant at 5% probability, ******= significant at 1% probability, *******= significant at 0.1%, ********= significant at 0.01% probability through F test, CD = critical difference, Days to 50% CI= Days to 50% curd initiation, Days to 50%CM= Days to 50% curd maturity, PH= Plant height, GPW= Gross plant weight, MCW= marketable curd weight, NCW = net curd weight, LL= leaf length, LW= leaf width, NoL= No of leaves, CL= curd length, CD= curd diameter, CoL= core length, CSI= curd size index, LSI= leaf size index, HI= harvest index, TMY= total marketable yield
