# Supplementary material for: Heterosis and combining ability in cytoplasmic male sterile and doubled haploid based Brassica oleracea progenies and prediction of heterosis using microsatellites
Source: PLoS One. 2019 Aug 19;14(8):e0210772. doi: 10.1371/journal.pone.0210772 (PMC6699688; doi:10.1371/journal.pone.0210772)
Supplement: S4 Table — *commercial standard checks. (DOCX) [file pone.0210772.s006.docx]

**S4 Table.** Characterization of parental CMS and DH lines including commercial checks for 16 agronomic traits

| **Parents/Traits** | **Days to 50% CI** | **Days to 50% CM** | **PH (cm)** | **GPW (g)** | **MCW (g)** | **NCW (g)** | **LL (cm)** | **LW (cm)** |
| --- | --- | --- | --- | --- | --- | --- | --- | --- |
| Ogu122-5A | 92.67 ± 0.58 | 123.00 ± 2.65 | 49.50 ± 3.52 | 2249.33 ± 94.24 | 1028.33 ± 64.49 | 618.33 ± 33.29 | 37.50 ± 1.00 | 19.33 ± 0.75 |
| Ogu115-33A | 92.00 ± 1.00 | 153.33 ± 1.53 | 35.33 ± 0.80 | 915.00 ± 142.57 | 555.00 ± 121.35 | 418.33 ± 33.29 | 31.63 ± 1.69 | 16.40 ± 1.01 |
| Ogu118-6A | 90.67 ± 1.53 | 133.00 ± 2.65 | 38.87 ± 1.97 | 1010.00 ± 52.92 | 730.00 ± 95.00 | 426.67 ± 20.82 | 37.20 ± 1.47 | 16.33 ± 1.26 |
| Ogu307-33A | 85.33 ± 1.53 | 118.00 ± 4.36 | 41.17 ± 4.07 | 1253.33 ± 75.06 | 633.33 ± 53.93 | 438.33 ± 24.66 | 41.37 ± 0.81 | 19.80 ± 1.54 |
| Ogu309-2A | 90.33 ± 1.53 | 125.00 ± 5.00 | 37.57 ± 4.42 | 855.00 ± 78.58 | 448.33 ± 37.53 | 286.67 ± 28.43 | 36.77 ± 1.48 | 17.13 ± 1.48 |
| Ogu33A | 92.00 ± 1.00 | 152.00 ± 2.00 | 42.87 ± 5.95 | 1660.00 ± 26.46 | 1105.67 ± 195.16 | 496.67 ± 36.86 | 38.00 ± 2.02 | 20.40 ± 1.05 |
| OguKt-2-6A | 92.00 ± 1.01 | 127.67 ± 3.51 | 43.60 ± 2.39 | 1536.33 ± 80.76 | 653.33 ± 27.54 | 403.33 ± 16.07 | 41.93 ± 1.63 | 21.37 ± 1.21 |
| Ogu1A | 92.00 ± 1.02 | 154.33 ± 2.08 | 42.07 ± 2.20 | 1203.00 ± 79.54 | 733.33 ± 56.86 | 340.00 ± 39.69 | 42.80 ± 2.52 | 18.80 ± 0.52 |
| Ogu13-85-6A | 89.00 ± 1.00 | 126.67 ± 3.21 | 36.93 ± 7.07 | 1063.00 ± 82.87 | 796.67 ± 132.51 | 415.00 ± 32.79 | 39.13 ± 2.12 | 23.60 ± 1.15 |
| Ogu1-6A | 90.00 ± 1.00 | 147.67 ± 2.52 | 43.43 ± 1.14 | 1223.33 ± 165.03 | 815.00 ± 77.62 | 498.33 ± 10.41 | 43.10 ± 2.51 | 21.13 ± 2.03 |
| Ogu2A | 97.67 ± 1.53 | 151.33 ± 3.21 | 37.70 ± 4.07 | 1241.67 ± 78.78 | 736.67 ± 38.84 | 435.00 ± 50.74 | 35.33 ± 1.04 | 18.37 ± 0.78 |
| OguKt-9-2A | 92.00 ± 1.00 | 135.67 ± 4.04 | 53.40 ± 3.54 | 1400.67 ± 88.12 | 925.00 ± 48.22 | 588.33 ± 22.55 | 49.27 ± 0.97 | 26.40 ± 1.85 |
| Ogu22-1A | 93.00 ± 1.00 | 153.00 ± 2.65 | 57.03 ± 1.29 | 1100.00 ± 47.70 | 583.33 ± 20.82 | 318.00 ± 32.79 | 52.57 ± 2.64 | 26.47 ± 1.68 |
| Ogu122-1A | 91.33 ± 1.53 | 153.33 ± 2.08 | 52.03 ± 1.59 | 1549.67 ± 84.50 | 970.00 ± 39.05 | 640.00 ± 35.00 | 50.80 ± 2.46 | 18.47 ± 1.00 |
| Ogu126-1A | 92.00 ± 1.00 | 153.00 ± 2.65 | 41.30 ± 3.65 | 1059.00 ± 81.07 | 455.00 ± 32.79 | 290.00 ± 22.91 | 34.53 ± 2.48 | 16.80 ± 0.61 |
| Ogu12A | 91.67 ± 1.53 | 153.67 ± 1.53 | 53.07 ± 1.76 | 1300.00 ± 156.20 | 663.33 ± 65.26 | 383.33 ± 32.53 | 38.43 ± 1.05 | 18.80 ± 0.61 |
| Ogu119-1A | 90.67 ± 1.53 | 122.67 ± 2.52 | 48.17 ± 4.51 | 1048.33 ± 82.51 | 420.00 ± 35.00 | 288.33 ± 18.93 | 37.17 ± 2.52 | 15.90 ± 0.46 |
| Ogu34-1A | 91.00 ± 1.00 | 129.00 ± 3.61 | 39.60 ± 3.77 | 1675.00 ± 82.61 | 930.00 ± 44.44 | 596.67 ± 88.08 | 47.47 ± 1.70 | 20.17 ± 0.67 |
| Ogu125-8A | 91.33 ± 1.53 | 123.33 ± 3.06 | 47.67 ± 3.56 | 1563.00 ± 122.50 | 1096.67 ± 68.07 | 718.33 ± 28.43 | 49.47 ± 3.56 | 27.13 ± 1.48 |
| Ogu33-1A | 89.33 ± 1.53 | 120.67 ± 2.52 | 43.17 ± 1.53 | 1166.67 ± 109.70 | 610.00 ± 39.69 | 388.33 ± 20.21 | 39.53 ± 1.05 | 21.17 ± 1.53 |
| DH-18-8-1 | 90.67 ± 1.53 | 144.67 ± 4.51 | 49.10 ± 1.57 | 1660.33 ± 113.18 | 1160.00 ± 78.58 | 670.00 ± 39.69 | 42.10 ± 2.51 | 20.80 ± 1.47 |
| DH-18-8-3 | 91.00 ± 1.00 | 154.00 ± 2.65 | 49.10 ± 2.07 | 1879.67 ± 106.40 | 923.33 ± 47.26 | 465.00 ± 61.44 | 49.40 ± 0.85 | 23.80 ± 1.57 |
| DH-53-1 | 92.67 ± 0.58 | 153.67 ± 1.53 | 53.27 ± 2.72 | 1798.33 ± 85.78 | 1231.67 ± 102.02 | 706.67 ± 33.29 | 38.83 ± 1.53 | 18.83 ± 1.53 |
| DH-53-6 | 92.00 ± 1.00 | 152.00 ± 2.65 | 51.00 ± 2.79 | 2008.33 ± 95.18 | 1107.00 ± 95.64 | 685.00 ± 27.84 | 50.70 ± 1.59 | 18.80 ± 1.54 |
| DH-53-9 | 94.67 ± 0.58 | 151.33 ± 3.21 | 44.33 ± 1.66 | 1931.67 ± 172.94 | 948.33 ± 40.10 | 516.67 ± 65.26 | 50.37 ± 1.85 | 22.60 ± 1.25 |
| DH-53-10 | 90.67 ± 1.53 | 152.67 ± 2.08 | 52.97 ± 2.68 | 1538.33 ± 55.30 | 758.33 ± 71.12 | 316.67 ± 20.82 | 44.83 ± 3.51 | 19.37 ± 1.21 |
| HVCF-29* | 90.33 ± 0.58 | 133.67 ± 1.53 | 44.93 ± 2.55 | 1345.00 ± 65.00 | 815.00 ± 32.79 | 453.33 ± 52.52 | 40.17 ± 2.52 | 19.47 ± 1.05 |
| HVCF-18* | 89.33 ± 1.53 | 142.33 ± 2.52 | 44.83 ± 4.30 | 1751.67 ± 60.07 | 855.00 ± 37.75 | 618.33 ± 43.11 | 40.03 ± 1.36 | 29.17 ± 6.35 |
| HVCF-16* | 91.00 ± 2.00 | 139.67 ± 2.08 | 51.73 ± 4.01 | 1103.33 ± 153.32 | 781.67 ± 27.54 | 451.67 ± 52.52 | 43.23 ± 2.05 | 23.50 ± 1.00 |
| Pahuja* | 90.00 ± 1.00 | 121.33 ± 3.51 | 30.83 ± 2.12 | 995.00 ± 27.84 | 548.33 ± 35.47 | 326.67 ± 27.54 | 28.47 ± 2.05 | 13.47 ± 1.00 |

*commercial standard checks

**S4 Table continue**

| **Parents/Traits** | **NoL** | **CL (cm)** | **CD (cm)** | **CoL (cm)** | **CSI (cm²)** | **LSI (cm²)** | **HI %** | **TMY (t/ha)** |
| --- | --- | --- | --- | --- | --- | --- | --- | --- |
| Ogu122-5A | 20.00 ± 2.00 | 9.27 ± 0.63 | 12.01 ± 1.32 | 4.28 ± 0.35 | 110.78 ± 4.54 | 725.43 ± 46.24 | 45.79 ± 3.91 | 41.13 ± 2.58 |
| Ogu115-33A | 16.33 ± 2.08 | 7.50 ± 0.33 | 9.88 ± 0.35 | 4.16 ± 0.13 | 74.09 ± 3.10 | 518.16 ± 28.40 | 60.25 ± 5.05 | 22.20 ± 4.85 |
| Ogu118-6A | 17.67 ± 2.52 | 8.41 ± 0.42 | 10.19 ± 0.56 | 4.08 ± 0.13 | 85.65 ± 5.58 | 607.88 ± 58.39 | 72.43 ± 10.24 | 29.20 ± 3.80 |
| Ogu307-33A | 23.00 ± 2.65 | 7.47 ± 0.20 | 9.27 ± 0.21 | 3.30 ± 0.30 | 69.19 ± 2.30 | 819.41 ± 71.54 | 50.52 ± 2.54 | 25.33 ± 2.16 |
| Ogu309-2A | 24.00 ± 1.00 | 7.53 ± 0.29 | 10.08 ± 0.47 | 3.18 ± 0.31 | 75.91 ± 3.24 | 631.27 ± 78.31 | 52.47 ± 0.76 | 17.93 ± 1.50 |
| Ogu33A | 18.67 ± 1.53 | 7.47 ± 0.29 | 9.85 ± 0.40 | 4.21 ± 0.11 | 73.56 ± 4.43 | 775.33 ± 58.94 | 66.50 ± 10.65 | 44.23 ± 7.81 |
| OguKt-2-6A | 16.67 ± 1.53 | 7.96 ± 0.28 | 10.65 ± 0.49 | 4.22 ± 0.13 | 84.79 ± 6.18 | 897.02 ± 80.00 | 42.54 ± 0.62 | 26.13 ± 1.10 |
| Ogu1A | 16.67 ± 1.53 | 8.82 ± 0.59 | 11.03 ± 0.43 | 4.72 ± 0.47 | 91.11 ± 2.72 | 803.95 ± 32.74 | 60.93 ± 1.48 | 29.33 ± 2.27 |
| Ogu13-85-6A | 23.67 ± 1.53 | 7.98 ± 0.50 | 10.22 ± 0.58 | 2.97 ± 0.13 | 81.72 ± 9.09 | 922.01 ± 15.27 | 74.62 ± 6.59 | 31.87 ± 5.30 |
| Ogu1-6A | 17.67 ± 2.52 | 8.18 ± 0.28 | 10.52 ± 0.46 | 3.38 ± 0.23 | 86.05 ± 2.44 | 908.27 ± 55.86 | 67.67 ± 12.59 | 32.60 ± 3.10 |
| Ogu2A | 18.33 ± 1.53 | 8.53 ± 0.37 | 11.82 ± 0.50 | 3.34 ± 0.30 | 100.99 ± 8.19 | 648.97 ± 33.68 | 59.48 ± 4.85 | 29.47 ± 1.55 |
| OguKt-9-2A | 16.00 ± 1.00 | 8.90 ± 0.63 | 12.58 ± 0.76 | 4.55 ± 0.26 | 112.28 ± 14.36 | 1301.82 ± 116.28 | 66.08 ± 1.79 | 37.00 ± 1.93 |
| Ogu22-1A | 16.33 ± 1.53 | 7.63 ± 0.28 | 9.75 ± 0.43 | 3.92 ± 0.12 | 74.38 ± 2.74 | 1389.29 ± 63.93 | 64.24 ± 21.71 | 28.13 ± 8.91 |
| Ogu122-1A | 16.33 ± 1.53 | 8.00 ± 0.48 | 9.83 ± 0.29 | 5.35 ± 0.09 | 78.59 ± 3.03 | 938.69 ± 77.90 | 62.78 ± 5.48 | 38.80 ± 1.56 |
| Ogu126-1A | 17.00 ± 1.73 | 7.67 ± 0.25 | 10.35 ± 0.46 | 3.55 ± 0.26 | 79.33 ± 3.64 | 579.51 ± 31.95 | 42.98 ± 0.36 | 18.20 ± 1.31 |
| Ogu12A | 15.00 ± 1.00 | 8.22 ± 0.28 | 10.27 ± 0.28 | 3.75 ± 0.10 | 84.38 ± 4.24 | 722.20 ± 13.57 | 51.14 ± 1.96 | 26.53 ± 2.61 |
| Ogu119-1A | 20.00 ± 2.00 | 7.68 ± 0.51 | 10.58 ± 0.42 | 4.02 ± 0.08 | 81.18 ± 4.71 | 590.18 ± 23.31 | 40.11 ± 2.65 | 16.80 ± 1.40 |
| Ogu34-1A | 27.67 ± 2.52 | 9.52 ± 0.28 | 13.52 ± 1.10 | 4.53 ± 0.43 | 128.46 ± 7.77 | 958.00 ± 65.30 | 55.63 ± 4.03 | 37.20 ± 1.78 |
| Ogu125-8A | 23.00 ± 2.65 | 8.71 ± 0.72 | 12.03 ± 0.18 | 3.86 ± 0.12 | 104.84 ± 9.37 | 1345.15 ± 160.16 | 70.52 ± 8.12 | 43.87 ± 2.72 |
| Ogu33-1A | 23.00 ± 2.65 | 8.88 ± 0.54 | 12.72 ± 1.17 | 3.32 ± 0.23 | 112.77 ± 9.02 | 836.07 ± 49.06 | 52.75 ± 7.84 | 24.40 ± 1.59 |
| DH-18-8-1 | 16.67 ± 1.53 | 9.08 ± 0.49 | 12.60 ± 0.61 | 4.10 ± 0.21 | 114.25 ± 0.88 | 873.23 ± 10.02 | 70.12 ± 7.19 | 46.40 ± 3.14 |
| DH-18-8-3 | 14.67 ± 2.52 | 8.52 ± 0.49 | 11.18 ± 0.31 | 3.29 ± 0.46 | 95.24 ± 5.95 | 1176.22 ± 91.29 | 49.28 ± 4.77 | 36.93 ± 1.89 |
| DH-53-1 | 14.67 ± 0.58 | 7.88 ± 0.63 | 10.26 ± 0.40 | 4.03 ± 0.08 | 81.05 ± 9.56 | 730.58 ± 50.18 | 68.63 ± 7.26 | 49.27 ± 4.08 |
| DH-53-6 | 18.67 ± 1.15 | 7.75 ± 0.40 | 9.90 ± 0.38 | 4.22 ± 0.25 | 76.82 ± 6.72 | 951.78 ± 54.56 | 55.35 ± 7.45 | 44.28 ± 3.83 |
| DH-53-9 | 19.33 ± 1.15 | 7.87 ± 0.18 | 10.42 ± 0.21 | 3.42 ± 0.15 | 81.93 ± 1.43 | 1139.83 ± 104.96 | 49.40 ± 5.65 | 37.93 ± 1.60 |
| DH-53-10 | 14.00 ± 1.00 | 8.27 ± 0.36 | 10.75 ± 0.20 | 4.39 ± 0.18 | 88.86 ± 2.59 | 871.05 ± 121.58 | 49.24 ± 3.15 | 30.33 ± 2.84 |
| HVCF-29* | 17.67 ± 2.52 | 8.73 ± 0.45 | 11.85 ± 0.69 | 4.42 ± 0.21 | 103.28 ± 0.98 | 782.57 ± 77.12 | 60.66 ± 2.93 | 32.60 ± 1.31 |
| HVCF-18* | 16.00 ± 1.00 | 9.77 ± 0.42 | 12.72 ± 0.61 | 4.85 ± 0.26 | 124.04 ± 1.64 | 1162.02 ± 210.79 | 48.80 ± 0.56 | 34.20 ± 1.51 |
| HVCF-16* | 20.00 ± 2.00 | 9.00 ± 0.45 | 12.58 ± 0.84 | 4.20 ± 0.09 | 113.06 ± 6.88 | 1017.32 ± 91.29 | 71.87 ± 11.18 | 31.27 ± 1.10 |
| Pahuja* | 16.00 ± 1.73 | 7.72 ± 0.15 | 10.55 ± 0.36 | 3.34 ± 0.10 | 81.41 ± 3.09 | 382.75 ± 29.77 | 55.19 ± 4.75 | 21.93 ± 1.42 |

*commercial standard checks
