# Supplementary material for: Heterosis and combining ability in cytoplasmic male sterile and doubled haploid based Brassica oleracea progenies and prediction of heterosis using microsatellites
Source: PLoS One. 2019 Aug 19;14(8):e0210772. doi: 10.1371/journal.pone.0210772 (PMC6699688; doi:10.1371/journal.pone.0210772)
Supplement: S5 Table — L: CMS lines; T: DH testers. (DOCX) [file pone.0210772.s007.docx]

**S5 Table** Estimates of phenotypic distance (PD), based on 16 phenotypic traits and genetic distance (GD), based on SSR, EST-SSRs molecular data, between parental lines and testers

| **Parents** | **T1** | | **T2** | | **T3** | | **T4** | | **T5** | | **T6** | |
| --- | --- | --- | --- | --- | --- | --- | --- | --- | --- | --- | --- | --- |
|  | **PD** | **GD** | **PD** | **GD** | **PD** | **GD** | **PD** | **GD** | **PD** | **GD** | **PD** | **GD** |
| **L1** | 3.80 | 0.94 | 5.20 | 0.94 | 5.30 | 0.96 | 5.20 | 0.96 | 5.40 | 0.95 | 5.10 | 0.95 |
| **L2** | 7.40 | 0.92 | 7.10 | 0.91 | 6.40 | 0.94 | 6.70 | 0.93 | 6.50 | 0.97 | 5.10 | 0.92 |
| **L3** | 5.49 | 0.91 | 6.08 | 0.91 | 5.45 | 0.93 | 5.91 | 0.93 | 6.03 | 0.97 | 4.52 | 0.92 |
| **L4** | 7.75 | 0.92 | 6.47 | 0.92 | 7.42 | 0.94 | 6.54 | 0.94 | 6.44 | 0.98 | 5.80 | 0.93 |
| **L5** | 8.27 | 0.89 | 7.25 | 0.88 | 8.01 | 0.91 | 7.58 | 0.90 | 6.74 | 0.95 | 6.01 | 0.89 |
| **L6** | 4.97 | 0.89 | 4.88 | 0.89 | 2.93 | 0.91 | 3.37 | 0.91 | 4.02 | 0.96 | 4.30 | 0.90 |
| **L7** | 5.84 | 0.89 | 4.14 | 0.88 | 5.74 | 0.91 | 4.89 | 0.90 | 4.01 | 0.95 | 2.94 | 0.89 |
| **L8** | 4.81 | 0.87 | 4.64 | 0.87 | 5.41 | 0.89 | 5.45 | 0.89 | 5.21 | 0.93 | 2.74 | 0.88 |
| **L9** | 6.23 | 0.83 | 5.81 | 0.83 | 6.55 | 0.85 | 6.47 | 0.85 | 5.64 | 0.90 | 6.03 | 0.84 |
| **L10** | 4.39 | 0.87 | 3.62 | 0.86 | 4.45 | 0.89 | 4.40 | 0.88 | 3.99 | 0.93 | 3.55 | 0.87 |
| **L11** | 5.82 | 0.79 | 5.82 | 0.78 | 6.03 | 0.81 | 6.55 | 0.80 | 5.16 | 0.85 | 5.44 | 0.79 |
| **L12** | 3.62 | 0.82 | 4.14 | 0.82 | 5.85 | 0.84 | 5.55 | 0.84 | 5.25 | 0.89 | 4.90 | 0.83 |
| **L13** | 7.12 | 0.75 | 4.60 | 0.74 | 6.54 | 0.77 | 5.65 | 0.76 | 4.65 | 0.81 | 4.59 | 0.75 |
| **L14** | 5.01 | 0.78 | 5.06 | 0.77 | 3.84 | 0.80 | 2.78 | 0.79 | 4.81 | 0.84 | 3.68 | 0.78 |
| **L15** | 7.70 | 0.74 | 6.24 | 0.74 | 7.02 | 0.76 | 6.87 | 0.76 | 6.05 | 0.80 | 4.41 | 0.75 |
| **L16** | 5.59 | 0.75 | 4.20 | 0.75 | 4.85 | 0.77 | 4.92 | 0.77 | 4.67 | 0.81 | 2.07 | 0.76 |
| **L17** | 7.84 | 0.75 | 6.79 | 0.75 | 7.55 | 0.77 | 7.05 | 0.77 | 6.67 | 0.82 | 4.62 | 0.76 |
| **L18** | 4.58 | 0.77 | 6.27 | 0.76 | 7.54 | 0.79 | 6.79 | 0.78 | 6.51 | 0.83 | 6.64 | 0.77 |
| **L19** | 4.09 | 0.52 | 4.89 | 0.52 | 6.03 | 0.54 | 5.45 | 0.64 | 5.11 | 0.69 | 6.47 | 0.63 |
| **L20** | 5.50 | 0.44 | 5.46 | 0.53 | 7.49 | 0.56 | 7.17 | 0.72 | 6.19 | 0.76 | 5.29 | 0.71 |

L: CMS lines; T: DH testers
