# Supplementary material for: Heterosis and combining ability in cytoplasmic male sterile and doubled haploid based Brassica oleracea progenies and prediction of heterosis using microsatellites
Source: PLoS One. 2019 Aug 19;14(8):e0210772. doi: 10.1371/journal.pone.0210772 (PMC6699688; doi:10.1371/journal.pone.0210772)
Supplement: S6 Table — * = significant at 5% probability, ** = significant at 1% probability, *** = significant at 0.1%, **** = significant at 0.01% probability through F test, MPH: Mid parent heterosis, BPH: better parent heterosis, SCA: specific combining ability (value in parenthesis). (DOCX) [file pone.0210772.s008.docx]

**S6 Table.** MPH of top ten crosses along with their BPH, mean performance and SCA effects (value in parenthesis) for 8 vegetative traits

| **Days to 50% curd initiation** | | | | **Days to 50% curd maturity** | | | |
| --- | --- | --- | --- | --- | --- | --- | --- |
| Cross combination | MPH% | BPH% | Mean performance | Cross combination | MPH% | BPH% | Mean performance |
| Ogu34-1A×DH-53-1 | -6.35** (-3.26***) | -7.19** | 86.00 | Ogu33A×DH-53-6 | -17.11** (-4.94***) | -17.11** | 126.00 |
| Ogu2A×DH-53-1 | -5.43** (-0.93) | -7.85** | 90.00 | Ogu33A×DH-53-1 | -15.81** (-2.64*) | -16.27** | 128.66 |
| Ogu2A×DH-53-9 | -5.37** (-0.45) | -6.83** | 91.00 | Ogu33A×DH-53-9 | -13.41** (-0.48) | -13.60** | 131.33 |
| Ogu33A×DH-53-1 | -4.69** (-1.65**) | -5.04** | 88.00 | Ogu33A×DH-18-8-1 | -12.58** (-3.29*) | -14.69** | 129.66 |
| Ogu309-2A×DH-53-9 | -4.50** (-2.72***) | -6.69** | 88.33 | Ogu22-1A×DH-53-10 | -12.54** (-13.58***) | -12.64** | 133.66 |
| Ogu125-8A×DH-53-1 | -3.99** (-1.93**) | -4.68** | 88.33 | Ogu1A×DH-18-8-3 | -10.27** (-11.61***) | -10.37** | 138.33 |
| Ogu307-33A×DH-18-8-3 | -3.59** (-5.44***) | -6.59** | 85.00 | Ogu33A×DH-18-8-3 | -10.24** (5.39***) | -10.82** | 137.33 |
| OguKt-2-6A×DH-53-9 | -3.57** (-0.73) | -4.93** | 90.00 | Ogu33A×DH-53-10 | -10.07** (5.97***) | -10.26** | 137.00 |
| Ogu1A×DH-53-9 | -3.57** (-0.61) | -4.93** | 90.00 | Ogu118-6A×DH-53-6 | -9.24** (-6.89***) | -14.91** | 129.33 |
| Ogu2A×DH-18-8-1 | -3.36** (-0.58) | -6.83** | 91.00 | Ogu2A×DH-18-8-3 | -8.52** (-10.06***) | -9.31** | 139.66 |
|  |  |  |  |  |  |  |  |
| **Plant Height (PH)** | | | | **Gross Plant Weight (GPW)** | | | |
| Cross combination | MPH% | BPH% | Mean Performance | Cross combination | MPH% | BPH% | Mean Performance |
| Ogu118-6A×DH-53-9 | 57.77** (11.18***) | 48.05** | 65.63 | Ogu118-6A×DH-53-10 | 170.14** (1018.24***) | 123.75** | 3442.00 |
| OguKt-2-6A×DH-53-9 | 54.21** (6.47***) | 52.93** | 67.80 | Ogu126-1A×DH-53-1 | 122.47** (800.31***) | 76.74** | 3178.33 |
| Ogu34-1A×DH-53-9 | 53.53** (8.96***) | 45.34** | 64.43 | Ogu307-33A×DH-18-8-3 | 121.64** (1172.05***) | 84.71** | 3472.00 |
| Ogu13-85-6A×DH-53-1 | 48.12** (13.61***) | 25.41** | 66.80 | Ogu115-33A×DH-53-9 | 118.03** (692.97***) | 60.66** | 3103.33 |
| OguKt-2-6A×DH-18-8-1 | 45.77** (5.59**) | 37.61** | 67.56 | OguKt-2-6A×DH-53-1 | 108.76** (569.98***) | 93.55** | 3480.66 |
| Ogu13-85-6A×DH-18-8-3 | 45.06** (11.18***) | 27.09** | 62.40 | Ogu307-33A×DH-18-8-1 | 107.02** (834.10***) | 81.65** | 3016.00 |
| Ogu307-33A×DH-18-8-3 | 44.98** (10.93***) | 33.27** | 65.43 | Ogu1-6A×DH-53-1 | 105.85** (849.37***) | 72.94** | 3110.00 |
| Ogu2A×DH-18-8-1 | 44.85** (9.28***) | 28.04** | 62.86 | Ogu22-1A×DH-53-6 | 104.61** (1175.32***) | 58.34** | 3180.00 |
| Ogu34-1A×DH-53-1 | 44.72** (8.99***) | 26.16** | 67.20 | Ogu1A×DH-53-9 | 99.19** (1022.31***) | 61.62** | 3122.00 |
| Ogu115-33A×DH-53-6 | 41.93** (7.58***) | 20.13** | 61.26 | Ogu115-33A×DH-53-1 | 98.53** (61.76) | 49.77** | 2693.33 |

*****= significant at 5% probability, ******= significant at 1% probability, *******= significant at 0.1%, ********= significant at 0.01% probability through F test, MPH: Mid parent heterosis, BPH: better parent heterosis, SCA: specific combining ability (value in parenthesis)

**S6 Table** **Continue**

| **Number of leaves (NoL)** | | | | **Leaf size index (LSI)** | | | |
| --- | --- | --- | --- | --- | --- | --- | --- |
| Cross combination | MPH% | BPH% | Mean performance | Cross combination | MPH% | BPH% | Mean Performance |
| OguKt-2-6A×DH-53-10 | 54.35** (5.53***) | 42.00** | 23.66 | Ogu126-1A×DH-53-1 | 155.50** (313.65***) | 129.09** | 1673.66 |
| Ogu115-33A×DH-53-10 | 51.65** (3.92***) | 40.82** | 23.00 | OguKt-2-6A×DH-53-1 | 144.80** (326.37***) | 122.09** | 1992.21 |
| Ogu1A×DH-53-6 | 42.27** (3.59**) | 23.21** | 23.00 | Ogu126-1A×DH-53-10 | 130.23** (397.38***) | 91.70** | 1669.81 |
| Ogu2A×DH-53-9 | 41.59** (5.36***) | 37.93** | 26.66 | Ogu115-33A×DH-53-6 | 118.91** (345.14***) | 169.04** | 1608.92 |
| Ogu118-6A×DH-53-10 | 41.05** (2.42*) | 26.42** | 22.33 | Ogu118-6A×DH-53-1 | 112.80** (70.98) | 94.93** | 1424.14 |
| Ogu33A×DH-53-1 | 40.00** (3.64**) | 25.00** | 23.33 | Ogu115-33A×DH-53-10 | 111.73** (139.55*) | 68.84** | 1470.71 |
| Ogu2A×DH-18-8-3 | 39.39** (3.64**) | 25.45** | 23.00 | Ogu307-33A×DH-53-10 | 99.43** (291.06***) | 93.52** | 1685.65 |
| Ogu12A×DH-53-10 | 37.93** (2.37*) | 33.33** | 20.00 | Ogu34-1A×DH-53-10 | 92.48** (524.64***) | 83.75** | 1760.28 |
| Ogu1A×DH-53-9 | 37.37** (2.31*) | 17.24** | 22.66 | Ogu34-1A×DH-53-1 | 90.16** (282.31***) | 67.59** | 1605.53 |
| Ogu122-1A×DH-53-10 | 35.48** (-2.36**) | 28.57** | 15.33 | Ogu118-6A×DH-53-10 | 89.85** (138.28*) | 61.17** | 1403.87 |
| **Leaf Length (LL)** | | | | **Leaf width (LW)** | | | |
| Cross combination | MPH% | BPH% | Mean Performance | Cross combination | MPH% | BPH% | Mean Performance |
| Ogu126-1A×DH-53-1 | 63.74** (4.77***) | 54.68** | 60.06 | OguKt-2-6A×DH-53-1 | 58.71** (2.47**) | 49.30** | 31.90 |
| OguKt-2-6A×DH-53-1 | 54.77** (4.92***) | 49.05** | 62.50 | Ogu126-1A×DH-53-10 | 56.87** (4.90***) | 46.47** | 28.36 |
| Ogu115-33A×DH-18-8-3 | 54.42** (8.01***) | 26.65** | 62.56 | Ogu126-1A×DH-53-1 | 56.22** (3.31***) | 47.79** | 27.83 |
| Ogu115-33A×DH-53-10 | 51.09** (4.11**) | 28.85** | 57.76 | Ogu118-6A×DH-53-1 | 56.21** (1.33) | 45.84** | 27.46 |
| Ogu13-85-6A×DH-53-1 | 49.38** (13.58***) | 48.81** | 58.23 | OguKt-2-6A×DH-53-9 | 53.15** (5.27***) | 48.97** | 33.66 |
| Ogu126-1A×DH-53-10 | 48.26** (5.72***) | 31.23** | 58.83 | Ogu115-33A×DH-53-6 | 53.03** (3.39***) | 43.26** | 26.93 |
| Ogu115-33A×DH-53-9 | 47.97** (6.00***) | 20.45** | 60.66 | Ogu34-1A×DH-53-10 | 47.39** (5.28***) | 44.46** | 29.13 |
| Ogu115-33A×DH-53-6 | 45.18** (7.14***) | 17.88** | 59.76 | Ogu118-6A×DH-53-10 | 46.97** (1.16) | 35.46** | 26.23 |
| Ogu125-8A×DH-53-1 | 40.58** (9.03***) | 25.47** | 62.06 | Ogu118-6A×DH-53-6 | 43.64** (1.01) | 34.22** | 25.23 |
| Ogu118-6A×DH-53-9 | 40.39** (10.71***) | 22.04** | 61.46 | Ogu307-33A×DH-53-10 | 42.47** (2.48**) | 40.91** | 27.90 |

*****= significant at 5% probability, ******= significant at 1% probability, *******= significant at 0.1%, ********= significant at 0.01% probability through F test, MPH: Mid parent heterosis, BPH: better parent heterosis, SCA: specific combining ability (value in parenthesis)
