# Supplementary material for: Heterosis and combining ability in cytoplasmic male sterile and doubled haploid based Brassica oleracea progenies and prediction of heterosis using microsatellites
Source: PLoS One. 2019 Aug 19;14(8):e0210772. doi: 10.1371/journal.pone.0210772 (PMC6699688; doi:10.1371/journal.pone.0210772)
Supplement: S7 Table — * = significant at 5% probability, ** = significant at 1% probability, *** = significant at 0.1%, **** = significant at 0.01% probability through F test, MPH: Mid parent heterosis, BPH: better parent heterosis, SCA: specific combining ability (value in parenthesis). (DOCX) [file pone.0210772.s009.docx]

**S7 Table.** MPH of top ten crosses along with their better parent heterosis and SCA effects (value in parenthesis) for 8 commercial traits

| **Core length (CoL)** | | | | **Curd length (CL)** | | | |
| --- | --- | --- | --- | --- | --- | --- | --- |
| Cross combination | MPH% | BPH% | Mean Performance | Cross combination | MPH% | BPH% | Mean Performance |
| Ogu122-1A×DH-53-6 | -30.31** (-0.88***) | -37.69** | 3.3 | Ogu119-1A×DH-18-8-1 | 36.24** (2.09***) | 25.69** | 11.41 |
| Ogu1A×DH-53-6 | -22.91** (-0.52**) | -27.00** | 3.4 | Ogu126-1A×DH-53-1 | 34.41** (0.75) | 32.56** | 10.45 |
| Ogu1A×DH-53-10 | -22.50** (-0.30) | -25.16** | 3.5 | Ogu122-1A×DH-53-10 | 33.09** (1.69***) | 30.91** | 10.82 |
| Ogu1A×DH-53-1 | -20.61** (-0.46*) | -26.36** | 3.4 | Ogu119-1A×DH-53-6 | 31.29** (0.77) | 30.67** | 10.12 |
| Ogu12A×DH-53-10 | -20.18** (-1.30***) | -26.02** | 3.2 | Ogu115-33A×DH-53-9 | 31.02** (0.74) | 27.97** | 10.06 |
| Ogu2A×DH-53-10 | -13.62** (-0.88***) | -24.20** | 3.3 | Ogu33A×DH-53-1 | 30.29** (0.45) | 26.85** | 10.00 |
| Ogu122-1A×DH-18-8-3 | -11.58** (0.13) | -21.87** | 4.2 | Ogu33A×DH-53-9 | 29.13** (0.39) | 25.85** | 9.90 |
| Ogu34-1A×DH-18-8-3 | -10.83** (-0.82***) | -23.09** | 3.4 | Ogu126-1A×DH-53-6 | 29.08** (0.24) | 28.39** | 9.95 |
| Ogu126-1A×DH-53-10 | -10.53** (-0.91***) | -19.12** | 3.5 | Ogu119-1A×DH-53-10 | 28.55** (0.90) | 23.94** | 10.25 |
| Ogu122-5A×DH-53-6 | 12.16* (0.62**) | 11.28 | 4.7 | Ogu115-33A×DH-53-6 | 27.65** (0.35) | 25.59** | 9.73 |
| **Curd diameter (CD)** | | | | **Curd Size index (CSI)** | | | |
| Cross combination | MPH% | BPH% | Mean Performance | Cross combination | MPH% | BPH% | Mean Performance |
| Ogu1-6A×DH-53-1 | 42.58** (1.40**) | 40.80** | 14.81 | Ogu122-1A×DH-53-10 | 84.33** (41.50***) | 73.67** | 154.33 |
| Ogu122-1A×DH-53-10 | 38.30** (1.96***) | 32.40** | 14.23 | Ogu126-1A×DH-53-1 | 80.34** (19.58**) | 78.43** | 144.61 |
| Ogu22-1A×DH-53-6 | 36.73** (1.67***) | 35.69** | 13.43 | Ogu119-1A×DH-18-8-1 | 73.32** (42.09***) | 48.23** | 169.36 |
| Ogu307-33A×DH-18-8-1 | 34.76** (1.72***) | 16.93** | 14.73 | Ogu1-6A×DH-53-1 | 73.01** (17.53*) | 67.98** | 144.55 |
| Ogu126-1A×DH-53-1 | 34.56** (0.97*) | 33.98** | 13.86 | Ogu33A×DH-53-1 | 72.36** (12.40) | 64.40** | 133.24 |
| Ogu115-33A×DH-53-6 | 34.46** (0.35) | 34.34** | 13.30 | Ogu119-1A×DH-53-10 | 72.05** (22.91**) | 64.60** | 146.27 |
| Ogu119-1A×DH-53-10 | 33.81** (1.21**) | 32.78** | 14.27 | Ogu115-33A×DH-53-6 | 70.76** (12.65) | 67.73** | 128.84 |
| Ogu2A×DH-53-6 | 33.71** (2.09***) | 22.84** | 14.52 | Ogu115-33A×DH-53-9 | 69.91** (14.55*) | 61.78** | 132.55 |
| Ogu1-6A×DH-53-6 | 33.51** (0.63) | 29.55** | 13.63 | Ogu119-1A×DH-53-6 | 68.05** (11.40) | 63.54** | 132.75 |
| OguKt-2-6A×DH-53-9 | 32.44** (1.13*) | 30.99** | 13.95 | Ogu2A×DH-53-6 | 66.85** (34.85***) | 46.88** | 148.33 |

*****= significant at 5% probability, ******= significant at 1% probability, *******= significant at 0.1%, ********= significant at 0.01% probability through F test, MPH: Mid parent heterosis, BPH: better parent heterosis, SCA: specific combining ability (value in parenthesis)

**S7 Table.** **continue**

| **Marketable curd weight (MCW)** | | | | **Net curd weight (NCW)** | | | |
| --- | --- | --- | --- | --- | --- | --- | --- |
| Cross combination | MPH% | BPH% | Mean Performance | Cross combination | MPH% | BPH% | Mean Performance |
| Ogu126-1A×DH-18-8-3 | 172.31** (676.08***) | 103.25** | 1876.66 | Ogu118-6A×DH-53-10 | 193.70** (332.68***) | 171.33** | 1157.66 |
| Ogu122-5A×DH-53-10 | 130.78** (731.04***) | 100.49** | 2061.66 | Ogu1A×DH-53-9 | 145.53** (415.99***) | 103.55** | 1051.66 |
| Ogu307-33A×DH-18-8-3 | 115.93** (584.02***) | 82.02** | 1680.66 | Ogu119-1A×DH-53-10 | 141.03** (222.18***) | 116.59** | 783.33 |
| Ogu119-1A×DH-53-10 | 104.24** (292.32***) | 58.68** | 1203.33 | Ogu126-1A×DH-18-8-3 | 139.29** (209.86***) | 94.27** | 903.33 |
| Ogu309-2A×DH-53-6 | 102.53** (527.68***) | 42.28** | 1575.00 | Ogu309-2A×DH-53-10 | 132.70** (0.41) | 108.57** | 754.33 |
| Ogu118-6A×DH-53-10 | 99.69** (344.98***) | 95.96** | 1486.00 | Ogu309-2A×DH-53-6 | 118.87** (337.52***) | 55.23** | 1063.33 |
| Ogu307-33A×DH-53-10 | 96.36** (238.04***) | 80.18** | 1366.33 | Ogu33-1A×DH-53-10 | 105.78** (270.79***) | 98.71** | 771.66 |
| Ogu1A×DH-53-9 | 93.86** (525.17***) | 71.88** | 1630.00 | Ogu309-2A×DH-18-8-3 | 101.86** (55.36) | 63.15** | 758.66 |
| Ogu309-2A×DH-53-10 | 93.81** (57.32) | 54.20** | 1169.33 | Ogu122-5A×DH-53-10 | 101.77** (280.79***) | 59.89** | 988.66 |
| Ogu33A×DH-53-1 | 92.76** (906.90***) | 82.90** | 2252.66 | Ogu13-85-6A×DH-18-8-3 | 101.67** (131.61***) | 90.82** | 887.33 |
| **Harvest index (HI)** | | | | **Total marketable yield (TMY)** | | | |
| Cross combination | MPH% | BPH% | Mean Performance | Cross combination | MPH% | BPH% | Mean Performance |
| Ogu122-5A×DH-53-9 | 56.64** (17.39***) | 50.91** | 74.56 | Ogu126-1A×DH-18-8-3 | 172.31** (27.04***) | 103.25** | 75.06 |
| Ogu126-1A×DH-18-8-3 | 49.16** (12.52***) | 39.61** | 68.80 | Ogu122-5A×DH-53-10 | 130.78** (29.24***) | 100.49** | 82.46 |
| Ogu12A×DH-18-8-3 | 41.99** (12.71***) | 39.41** | 71.29 | Ogu307-33A×DH-18-8-3 | 115.93** (23.36***) | 82.02** | 67.22 |
| Ogu119-1A×DH-53-10 | 35.84** (10.63***) | 23.25** | 60.69 | Ogu119-1A×DH-53-10 | 104.24** (11.69***) | 58.68** | 48.13 |
| Ogu126-1A×DH-53-10 | 29.08** (3.61) | 20.86** | 59.51 | Ogu309-2A×DH-53-6 | 102.53** (21.11***) | 42.28** | 63.00 |
| Ogu122-5A×DH-53-10 | 23.63** (3.16) | 19.30** | 58.74 | Ogu118-6A×DH-53-10 | 99.69** (13.80***) | 95.96** | 59.44 |
| Ogu122-1A×DH-53-10 | 22.52** (17.08***) | 9.31 | 68.62 | Ogu307-33A×DH-53-10 | 96.36** (9.52***) | 80.18** | 54.65 |
| Ogu33A×DH-53-1 | 21.79** (33.46***) | 19.90** | 82.29 | Ogu1A×DH-53-9 | 93.86** (21.01***) | 71.88** | 65.20 |
| Ogu309-2A×DH-53-6 | 21.64** (9.07**) | 18.47** | 65.57 | Ogu309-2A×DH-53-10 | 93.81** (2.29) | 54.20** | 46.77 |
| Ogu22-1A×DH-53-9 | 21.16** (12.81***) | 7.17 | 68.84 | Ogu33A×DH-53-1 | 92.76** (36.28***) | 82.90** | 90.10 |

*****= significant at 5% probability, ******= significant at 1% probability, *******= significant at 0.1%, ********= significant at 0.01% probability through F test, MPH: Mid parent heterosis, BPH: better parent heterosis, SCA: specific combining ability (value in parenthesis)
